# Tumor-Derived Lactic Acid Modulates Activation and Metabolic Status of Draining Lymph Node Stroma

**DOI:** 10.1158/2326-6066.CIR-21-0778

**Published:** 2022-03-09

**Authors:** Angela Riedel, Moutaz Helal, Luisa Pedro, Jonathan J. Swietlik, David Shorthouse, Werner Schmitz, Lisa Haas, Timothy Young, Ana S.H. da Costa, Sarah Davidson, Pranjali Bhandare, Elmar Wolf, Benjamin A. Hall, Christian Frezza, Thordur Oskarsson, Jacqueline D. Shields

**Affiliations:** 1 Mildred Scheel Early Career Centre, University Hospital of Würzburg, Würzburg, Germany.; 2 MRC Cancer Unit, Hutchison/MRC Research Centre, University of Cambridge, Cambridge, United Kingdom.; 3 The Heidelberg Institute for Stem Cell Technology and Experimental Medicine (HI-STEM gGmbH), Heidelberg, Germany.; 4 Division of Stem Cells and Cancer, German Cancer Research Center (DKFZ), Heidelberg, Germany.; 5 Institute for Biochemistry and Molecular Biology, Theodor Boveri Institute, University of Würzburg, Würzburg, Germany.; 6 Department of Medicine, University of Cambridge, Cambridge, United Kingdom.; 7 Cancer Systems Biology Group, Theodor Boveri Institute, University of Würzburg, Würzburg, Germany.

## Abstract

Communication between tumors and the stroma of tumor-draining lymph nodes (TDLN) exists before metastasis arises, altering the structure and function of the TDLN niche. Transcriptional profiling of fibroblastic reticular cells (FRC), the dominant stromal population of lymph nodes, has revealed that FRCs in TDLNs are reprogrammed. However, the tumor-derived factors driving the changes in FRCs remain to be identified. Taking an unbiased approach, we have shown herein that lactic acid (LA), a metabolite released by cancer cells, was not only secreted by B16.F10 and 4T1 tumors in high amounts, but also that it was enriched in TDLNs. LA supported an upregulation of Podoplanin (Pdpn) and Thy1 and downregulation of IL7 in FRCs of TDLNs, making them akin to activated fibroblasts found at the primary tumor site. Furthermore, we found that tumor-derived LA altered mitochondrial function of FRCs in TDLNs. Thus, our results demonstrate a mechanism by which a tumor-derived metabolite connected with a low pH environment modulates the function of fibroblasts in TDLNs. How lymph node function is perturbed to support cancer metastases remains unclear. The authors show that tumor-derived LA drains to lymph nodes where it modulates the function of lymph node stromal cells, prior to metastatic colonization.

## Abstract (precis)

How lymph node function is perturbed to support cancer metastases remains unclear. The authors show that tumor-derived lactic acid drains to lymph nodes where it modulates the function of lymph node stromal cells, prior to metastatic colonization.

## Introduction

The lymphatic system and lymph nodes are an integral part of the adaptive immune system and critical for effective immune responses. Many tumors exploit lymphatic vessels to spread and colonize downstream lymph nodes, which is an indicator of poor prognosis (1–3). For successful tumor growth and metastasis, establishment of a protumorigenic environment is necessary (4). As part of this, stromal components are manipulated by direct cell–cell interaction with tumor cells and by secreted factors. The range of bioactive molecules secreted by tumor cells is diverse, including proteins and peptides (5, 6), lipids (7, 8), nucleic acids (9, 10), exosomes or vesicles (11, 12), and low molecular weight metabolites such as lactate (13–15). Tumors also may use these bioactive factors to prime the metastatic niche environment even before the arrival of cancer cells at the distant site (16).

Compared with normal tissue-resident cells, tumor cells exhibit an altered metabolic profile, typically becoming more glycolytic, increasing glucose uptake and lactate secretion (17). As a consequence, tumors often contain high concentrations of lactic acid (LA; ref. 18). Although originally considered a waste product, it is now accepted that most cell types can take up and use lactate to fuel respiration (13, 19–22). An increasing body of evidence shows lactate as a key participant in numerous metabolic pathways, even acting in a signaling capacity (23–26). In tumors, lactate may act both on tumor cells and supporting stromal cells (13, 27–29). Lactate treatment has been reported to induce hypoxia-inducible factor-1α (Hif1α)-driven M2-like polarization of macrophages and concurrent expression of vascular endothelial growth factor (13). Similarly, lactate uptake in normoxic endothelial cells leads to Hif1α-driven angiogenesis (28), and cancer-associated fibroblasts (CAF) can take up and utilize lactate (30). Additionally, lactic acid can blunt immune responses at the primary tumor and in lung metastases by suppressing T- and natural killer (NK)-cell activation while supporting proliferation and suppressor function of regulatory T cells (31–33). High lactate levels also are associated with metastasis, tumor recurrence, and poor survival in cervical cancer (34). Furthermore, lactate dehydrogenase A (LDHA) expression is upregulated in many cancers where it is associated with more aggressive disease (35) and survival of cancer-initiating cells (36). In mouse models, targeting LDHA inhibits tumor growth and leads to delayed metastasis formation (36–38). Together, these data highlight that metabolic reprogramming not only impacts tumor cells themselves, but also affects adjacent stromal cells and subsequent metastasis formation.

We previously demonstrated that fibroblastic reticular cells (FRC) in TDLNs immediately downstream of murine melanoma, undergo remodeling and reprogramming prior to metastatic spread (39). FRCs are key for lymph node microarchitecture and also function as immune mediators. Transcriptional profiling of FRCs in TDLNs has revealed changes in immune-related pathways including downregulation of IL7 and CCL21, and changes in activation state, as measured by upregulation of CAF markers including podoplanin (Pdpn) and Thy1. These data also predicted an altered function of FRC mitochondria (39). Despite the observation that FRCs in TDLNs are functionally altered, the drivers of the changes observed remained undetermined; candidates included mechanical stresses created by elevated fluid drainage, tumor-derived cytokines, metabolites, or exosomes. Here, we propose tumor-derived LA is a driving factor in preconditioning of FRCs of premetastatic TDLNs, promoting a change in their metabolic phenotype via alterations to mitochondrial function, induction of CAF-like characteristics and adaptations to their immunologic function. Using computational models, pH was predicted as a key driver of metabolic changes, and a synergistic relationship between lactate and protons as the cause of subsequent transformation measured in TDLNs. Consistent with the modeling data, tumor-derived metabolites decreased intracellular pH leading to increased expression of activation markers Pdpn and Thy1 and impacted mitochondrial behavior of FRCs *in vitro* and *in vivo*.

## Materials and Methods

### Animal experiments

All animal experiments using immune competent 8- to 9-week-old female C57BL/6 mice (Charles River Laboratories) were performed in accordance with UK Home Office regulations under Project License PPL 80/2574. Conditioned medium, RPMI (Sigma-Aldrich, #R8758), PBS (Sigma-Aldrich, #D8537), or 15 mmol/L LA (Sigma-Aldrich, #L6402) in RPMI were injected subcutaneously into the shoulder region for 11 days every day and animals were euthanized and lymph nodes retrieved on day 11. For syngeneic, orthotopic tumors, 2.5 × 10^5^ B16.F10 melanoma cells were inoculated subcutaneously into the shoulder region and animals were euthanized and tumors and draining lymph nodes retrieved at day 11.

Experiments involving the injection of the 4T1 cell line into mice to induce breast tumors were approved by the governmental review board of the state of Baden-Wuerttemberg, Regierungspraesidium Karlsruhe, Germany under the authorization number G-65/17 and were according to the German legal regulations. For sham control animals, in which no experimental procedures were performed and only organs were taken as controls, the authorization number was DKFZ356. Animals included were immune competent 7- to 10-week-old female BALB/c mice (Janvier Labs). To induce breast tumors, 2 × 10^3^ 4T1 murine breast cancer cells were injected into the fourth mammary fat pad and animals were euthanized and tumors and draining lymph nodes retrieved at day 14.

Conditioned medium from cultures of ^13^C_6_-labeled (Sigma, #389374) 4T1 tumor cells or RPMI was injected subcutaneously into the shoulder region for 11 days every day using immune competent 7- to 10-week-old female BALB/c mice. Animals were euthanized and lymph nodes retrieved on day 11. This experiment was approved by the governmental review board of the state of Bayern, Regierung von Unterfranken, under the authorization number 55.2.2–2532–1242–12 and was according to the German legal regulations.

Animals were excluded if tumors failed to form or if health concerns were reported. Tumor size was monitored with calipers and the volume was calculated based on the ellipsoid formula π/6 × (length × width^2^).

### Flow cytometry

Extracted lymph nodes from mice were mechanically disrupted and digested in a 500-µL mixture of 1 mg/mL collagenase A and D (Roche, #10103578001, #11088858001) and 0.4 mg/mL DNase I (Roche, #10104159001) in PBS at 37°C for 30 to 60 minutes with 600 rpm rotation in a thermo-mixer (Eppendorf). EDTA (final concentration 10 mmol/L) was then added and cells were passed through a 70-µm mesh prior to immunostaining. *In vitro* cultured cells were detached by either Trypsin-EDTA (Gibco, Life Technologies, #25200056) or Accutase (Sigma-Aldrich, #A6964). Cells were stained with fixable viability dye live/dead violet (Molecular Probes, #L34958) and combinations of the following fluorescently conjugated antibodies: Pdpn (clone #8.1.1), CD45 (clone #30-F11), and CD31 (clone #MEC13.3; all BioLegend). For intracellular staining the FoxP3/Transcription Factor Fixation/Permeabilization Kit (eBioscience, #00–5523–00) was used and the manufacturer's guidelines were followed.

*In vitro* cultured FRCs were stained with Pdpn (clone #8.1.1), Thy-1 (clone #53–2.1), and Ki67 (clone #11F6; all BioLegend).

For mitochondrial staining, cells were stained with MitoTracker Green FM (MTG; Cell Signaling Technology, #9074) and Tetramethylrhodamine, Methyl Ester, Perchlorate (TMRM; Thermo Fisher Scientific, #T668) for 30 minutes at 37°C and 5% CO_2_. Staining was performed with 50 nmol/L MTG and 20 nmol/L TMRM in 100% conditioned media, the same RPMI medium containing 15 mmol/L LA, sodium lactate (resuspended in water; Sigma-Aldrich, #71716) or an equal amount of water (Veh – H_2_O), or medium adjusted to pH6 by the use of hydrochloric acid (HCl). All media was supplemented with 2% FBS (Sigma-Aldrich, #12103C) and 100 U/mL penicillin-streptomycin (Sigma-Aldrich, #15140122).

Intracellular pH was measured using a cell-permeant ratiometric fluorescent pH indicator called SNARF [SNARF-5F5-(and-6)-carboxylic acid, acetoxymethyl ester, acetate; Thermo Fisher Scientific, #S23923]. This SNARF pH indicator is excitable at 532 nm and exhibits a pH-dependent emission shift from 580 nm to 640 nm. The cells were stained with 10 nmol/L SNARF in treatment/conditioned medium (as for MTG and TMRM) for 30 minutes at 37°C and 5% CO_2_ and analyzed using an LSR Fortessa with a 532 laser and 586/15 and 620/20 filters. Emission shifts could then be calculated based on λ_586_/λ_610_ using the geometric mean with lower intracellular pHs giving higher values.

All flow cytometry was performed on LSR Fortessa (BD Biosciences) analyzers and offline analysis was performed with FlowJo software (Treestar, version 10.7.2).

### Cell sorting followed by RNA analysis

For RNA processing, cell sorting was performed on a high-speed Influx Cell Sorter or a FACSAria (100 µmol/L nozzle, both BD Biosciences) into RNA protect Cell Reagent (QIAGEN, #76526). Lymph node FRCs cell suspensions were sorted based on expression of Pdpn (clone #8.1.1) within the CD45 (clone #30-F11), and CD31 (clone #MEC13.3; all BioLegend) negative gate. RNA was then isolated with the RNeasy plus micro Kit (QIAGEN, #74034) or the ARCTURUS PicoPure RNA Isolation Kit (Thermo Fisher Scientific, #KIT0204) and RNA quality and quantity was analyzed with a Bioanalyzer (Agilent Technologies). Only RNA samples with a RNA integrity number (RIN) above 8 and total concentration of 100 pg/µL were further processed for whole transcriptome amplification via the Ovation PicoSL WTA V2 Kit (NuGEN, #3312). qRT-PCR using 20 ng cDNA input material was performed using TaqMan assays with the following primers/probes (all Applied Biosystems): *Pdpn* Mm01348912_g1, *Thy1* Mm00493682_g1, *Il7* Mm01295803_m1, and housekeeping gene *Actb* Mm00607939_s1. qRT-PCR was performed on a StepOne or ViiA 7 Real Time PCR System instrument in a relative quantification setting (both Life Technologies). Gene expression level are shown as 2^–ddCt^.

### Cell culture

B16.F10 (CRL 6475, ATCC, acquired in 2011) and 4T1 (a gift of T. Oskarsson) cells were maintained in DMEM (Gibco, #41966) with 10% FBS and 100 U/mL penicillin-streptomycin. An FRC cell line was generated from lymph nodes of p53^ER/ER^ C57BL/6 mice and characterized based on the expression levels of Pdpn and VCAM-1 and the lack of expression of CD45 and CD31 (39). All cell lines were used within a 15-passage window and tested negative for *Mycoplasma*. They have not been reauthenticated in the past year. FRCs were cultured in RPMI medium supplemented with 10% FBS and 100 U/mL penicillin-streptomycin, 10 mmol/L HEPES (Sigma-Aldrich, #SRE0065), and 15 µmol/L β-mercaptoethanol (Sigma-Aldrich, #M3148). All cells were incubated at 37°C with 5% CO_2_.

For tumor cell–conditioned medium (TCM) or control conditioned medium (CCM) production; B16.F10 cells, 4T1 cells, or FRCs were seeded into 175 cm^2^ culture flasks at 20% confluency in their normal growth medium, and after 24 hours the medium was exchanged for RPMI without supplements. Twenty-four hours later, the medium was retrieved and filtered (pore size 0.22 µm) and frozen at −80°C for storage. Conditioned media was never subject to repeated freezing/thawing.

For TCM and CCM production including the Ldha/Ldhb inhibitor GSK2837808A (GlaxoSmithKline; Tocris, #5189); cells were seeded as above, but with various concentrations of the inhibitor in the medium or the same volume of DMSO (vehicle control). Thereafter, lactate levels were measured as described below (Lactate measurements) and 30 µmol/L inhibitor was added in the medium for further experiments.

For studies assessing the effects of various factors on FRCs, 5,000 FRCs were seeded onto a six-well plate or 35,000 FRCs were seeded into 25 cm^2^ culture flasks in RPMI medium supplemented with 10% FBS and 100 U/mL penicillin-streptomycin, 10 mmol/L HEPES, and 15 µmol/L β-mercaptoethanol (full growth medium). Twenty-four hours later, medium was exchanged for 100% conditioned media, RPMI containing 15 mmol/L LA, sodium lactate (both resuspended in water) or an equal amount of water (Veh – H_2_O), RPMI adjusted to pH6 by using HCl, or RPMI containing recombinant protein (TGFβ 2ng/mL; RnD Systems, #7666-MB-005/CF), Osteopontin (5 µg/mL; RnD Systems, #441-OP-050/CF), or Angiopoietin2 (1 µg/mL; RnD Systems, #7186-AN-025/CF). All were supplemented with 2% FBS and 100 U/mL penicillin-streptomycin. After 48 hours each medium was refreshed and after 96 hours or 168 hours the cells were harvested for analyses.

For FRC treatments including GSK2837808A, 20,000 FRCs were seeded onto a six-well plate in full growth medium. Twenty-four hours later the medium was exchanged for 100% conditioned media with GSK2837808A (the inhibitor was still present in the medium after TCM and CCM production), supplemented with 10% FBS and 100 U/mL penicillin-streptomycin. After 48 hours the medium was exchanged for the same conditioned medium and after 96 hours the cells were harvested for analyses.

RNA extraction was performed using RNeasy Mini Kit (QIAGEN, #74104). One microgram of RNA was used for reverse transcription using First Strand cDNA synthesis Kit (Thermo Fisher Scientific, #K1612). Thereafter, qRT-PCR was performed using 20 ng cDNA input material in triplicates using the TaqMan assays with the following primers/probes (all Applied Biosystems): *Pdpn* Mm01348912_g1, *Thy1* Mm00493682_g1, *Il7* Mm01295803_ m1, *Mct1* Mm01306379_m1, *Mct4* Mm00446102_m1, *Ldha* Mm01612132_g1, and the housekeeping gene *Actb* Mm00607939_s1. The qRT-PCR was performed on a StepOne or ViiA 7 Real Time PCR System instrument in a relative quantification setting (both Life Technologies). Gene expression level are shown as 2^–ddCt^.

### RNA sequencing

#### Construction of sequencing libraries

Total RNA was isolated using the RNeasy Mini Kit (QIAGEN). The concentration, integrity, and purity was determined by NanoDrop (Thermo Fisher Scientific) and BioAnalyzer (Agilent). The NEBNext Poly(A) mRNA Magnetic Isolation Module (New England Biolabs, #E7490) was used for 500 ng. Thereafter, libraries for RNA sequencing (RNA-seq) were prepared according to the instructions for the NEBNext Ultra II Directional RNA Library Prep Kit for Illumina (New England Biolabs, #E7760) with 10 PCR cycles for amplification and sequenced on an Illumina NextSeq500 platform. FASTQ generation was carried out using CASAVA.

#### Quality control and alignment

FASTQ files were evaluated for quality using FASTQC (Babraham Bioinformatics - FastQC A Quality Control tool for High Throughput Sequence Data). Subsequently, the reads were aligned to ENSEMBL GRCm38 Mus Musculus's genome version 100 using STAR (2.7.0a) applying the default parameters, an average of 10 million reads were uniquely mapped per sample. FeatureCounts was used to quantify the reads that were mapped to each gene and to generate count matrices (40).

#### Differential gene expression and gene enrichment analysis

Differential gene expression and gene enrichment analyses were performed using the DEseq2 R package (41). To exclude low quality genes, genes that were detected in less than three samples and those that had less than 10 reads were removed. After these criteria, a total of 12,137 genes remained for further analysis. Differentially expressed genes (DEG) were determined between conditions by applying DESeq function provided by DESeq2 (41). Benjamini–Hochberg was the method of choice to obtain adjusted *P* values for multiple testing.

#### Heatmaps and plots

Heatmaps were generated using ggplot2 and pheatmap packages in R with default settings.

#### Pathway analyses

Enrichment analysis was conducted using Metascape (42). A gene list with the up- and/or downregulated significant deregulated genes [log_2_ fold change (FC) > 0.39 or < − 0.39, adjusted *P* > 0.05) was submitted to Metascape web portal. The pathway and process enrichment were initiated with a minimum overlap of three genes, a *P* value cut-off point of 0.01, and a minimum enrichment of 1.5. The results were downloaded and visualized using GraphPad prism.

#### Transcription factor analyses

For transcription factor (TF) analyses the webtool Enrichr was used (43, 44). A gene list with all significant deregulated genes (log_2_ FC > 0.39 or < - 0.39, adjusted *P* > 0.05) was submitted. For the shown TFs, the output for chromatin immunoprecipitation (ChIP) Enrichment Analysis (ChEA) 2016 was chosen.

### ELISA

Protein quantification per ELISA was performed according to the R&D System Quantikine IL7 ELISA Kit (R&D Systems, #M70000,) product manual.

### Confocal microscopy

Five hundred FRCs were seeded on eight chamber glass slides and treated over different periods of time. Chamber and tissue slides were fixed in either 4% PFA for 10 minutes at room temperature or in 1:2 acetone:methanol solution for 5 minutes at −20°C. Cells were permeabilized with 0.2 Triton X-100 (Sigma-Aldrich, #X100) in PBS and incubated for 5 minutes on ice. Followed by a blocking step for 40 minutes in 5% chicken serum (Sigma-Aldrich, #C5405), 2% BSA (Sigma-Aldrich, A9418), 0.1% Tween20 (Sigma-Aldrich, #P1379) in PBS at room temperature. Finally, the cells were incubated with the following primary antibodies specific for the indicated mouse antigens at 4°C overnight—Collagen I (Abd Serotec, #2150–1410), Hif1a (clone #ERP16897, Abcam), Ldha (Abcam, #ab52488), CD31 (clone #MEC13.3, BioLegend), Pdpn (clone #8.1.1, BioLegend)—and DAPI nuclear counterstain (Thermo Fisher Scientific, #62248). Samples were washed and incubated with the appropriate Alexa Fluor-conjugated secondary antibody (Thermo Fisher Scientific, #A-21442, #A-21441, #A-21451, #A-21471), then slides were mounted in ProLong Gold (Thermo Fisher Scientific, #P36930). For live cell imaging of mitochondria, FRCs were stained with Hoechst 33342 (Invitrogen, #H3570) and 50 nmol/L MTG for 30 minutes at 37°C and 5% CO_2_ and imaged live after a washing step in RPMI medium with the respective treatment. All confocal images were taken using either a Leica SP5 or Zeiss LSM 880 confocal microscope and processed with Volocity (Perkin Elmer) or FIJI (ImageJ).

### Metabolic assays

Oxygen consumption rate (OCR) and extracellular acidification rate (ECAR) were measured using an Extracellular Flux Analyzer, Seahorse XF^e^14, system (Seahorse Bioscience). Thirty-thousand FRCs were seeded into each well of a 24-well plate, incubated at 37°C and 5% CO_2_ overnight and directly before the assay the medium was changed to bicarbonate-free RPMI (Agilent, #103576–100) and, where appropriate, bicarbonate-free RPMI with 15 mmol/L LA or conditioned medium. Cells were then incubated in a CO_2_-free incubator at 37°C for 30 minutes. OCR and ECAR were determined under basal conditions and in response to 1 µmol/L oligomycin, 1 µmol/L fluoro-carbonyl cyanide phenylhydrazone (FCCP), and 1 µmol/L rotenone + 1 µmol/L antimycin A (Agilent, all part of kit #103010–100). Protein content (BCA assay, Thermo Fisher Scientific, #23227) was used for data normalization.

### PCR array

The RT^2^ Profiler PCR Array PAMM-120ZG (Mouse Fibrosis, QIAGEN, #330231) was used. RNA was extracted using a RNeasy Mini Kit. Four hundred nanograms of total RNA was then reverse transcribed using the RT^2^ First Strand Kit (QIAGEN), followed by real-time PCR using SYBR Green technology (QIAGEN, all part of the RT^2^ Profiler PCR Array kit) and a Roche LightCycler 480. Each gene was normalized on recommended housekeeping genes and FCs were calculated based on sample replicates.

### Lactate measurements

L-Lactate was measured in conditioned medium and tissue samples using the L-Lactate Assay kit (abcam, #ab65331) according to the manufacturer's instructions. All samples were deproteinized with the Deproteinizing Sample Preparation Kit–trichloroacetic acid (TCIA; abcam, #ab204708) before assaying.

Tissue samples were weighed into Precellys tubes prefilled with ceramic beads (Bertin Instruments, #P000911-LYSKO-A). The exact 6x volume (1 mg tissue/6 µL buffer) of Lactate Assay Buffer was added on ice and samples were lysed using a Precellys 24 homogeniser (Bertin Instruments).

### Sulforhodamine B colorimetric cell-density assay

Cells were fixed in 1% (or 10%) TCIA [Sigma-Aldrich, #T9159; volume for volume (v/v)] in H_2_O for 30 minutes and stained with 0.057% (w/v) sulforhodamine B (SRB) stain (Sigma-Aldrich, #S1402) in 1% (v/v) TCIA for 30 minutes. Plates were then washed three times in 1% (v/v) TCIA and dried overnight. Finally, the dried cell debris were resuspended in 10 mmol/L Tris base (Sigma-Aldrich, #TRIS-RO) and optical density was measured at 510 nm in a plate reader (Tecan).

#### Exosome isolation/depletion by ultracentrifugation

To remove cells and cellular debris, conditioned medium of B16.F10 cells was centrifuged for 20 minutes at 2,000 × g and 4°C. The supernatant was recovered and transferred into polyallomer tubes suitable for ultracentrifugation. Following centrifugation for 30 minutes at 10,000 × g and 4°C, the supernatant was transferred to a fresh tube and centrifuged for 70 minutes at 100,000 × g and 4°C. The supernatant represented exosome-free medium and was removed leaving 2 mm of liquid above the pellet. To wash the isolated exosomes, the pellet was resuspended in 1 mL PBS. Then PBS was added to fill the tube completely. After centrifugation for 1 hour at 100,000 × g and 4°C, the supernatant was removed and the pellet was resuspended in PBS and stored at −80 °C.

### Depletion of factors in conditioned medium

#### Heat denaturation

Conditioned media were incubated at 99°C for 15 minutes.

#### Freeze–thaw cycles

Conditioned media were frozen at −80°C for 15 minutes and thawed at 60°C for 15 minutes. This was repeated three times.

#### Filtering

Conditioned media were filtered at 4,000 × g and 4°C using Amicon Ultra-4 Centrifugal Filter Units (Merck Millipore, #UFC800308) with a membrane with a molecular weight cut-off (MWCO) of 3 kDa. Filtering of 4 mL medium was proceeded until 100 µL medium was left above the filter. Supernatant and filtrate were recovered and filled up to the initial volume with RPMI.

#### Benzonase treatment

Benzonase (Sigma-Aldrich, #E8263) was added to conditioned media with a final concentration of 1 U/µL. After that, media were incubated for 30 minutes at 37°C.

#### DNase I treatment

DNase I (Roche, #10104159001) was added to conditioned media with a final concentration of 2.5 U/mL. After that, media were incubated for 30 minutes at 37°C. DNase I was inactivated by heating at 75°C for 10 minutes.

### Isotopic flux analysis

4T1 murine tumor cells were seeded at 30% confluency and after 24 hours, normal growth medium was replaced by RPMI medium with 10 mmol/L ^13^C_6_-D-glucose (Sigma-Aldrich, #389374) without FBS. After 24 hours, the conditioned medium was obtained and sterile filtered. This medium was subsequently used in *in vivo* experiments or in *in vitro* experiments to stimulate FRCs for 4 days as mentioned above (Cell culture) and analyzed using LC–MS.

FRCs were seeded (1 × 10^4^) onto six-well plates. After 24 hours, the normal growth medium was exchanged for RPMI medium with 5 mmol/L ^13^C_3_-L-LA (LGC Standards, TRC-L113507–10MG) and 2% FBS, which was titrated to pH 6.75 with 1M HCl. After 48 hours of culture, the medium was replaced with RPMI medium with 5 mmol/L ^13^C_3_-L-LA and 2% FBS. After 48 hours, the cells were harvested and prepared for LC–MS.

### Sample preparation for LC–MS analysis

Lymph nodes were weighed into Precellys tubes prefilled with ceramic beads. An exact volume of metabolite extraction solution (30% acetonitrile, Sigma-Aldrich, #1000291000; 50% methanol, Sigma-Aldrich, #439193; and 20% water alternatively 80% methanol and 20% water) was added to obtain 60 mg specimen per milliliter of extraction solution. Samples were lysed using a Precellys 24 homogeniser. The suspension was mixed and incubated for 15 minutes at 4°C in a Thermomixer (Eppendorf), followed by centrifugation (16,000 × g for 15 minutes at 4°C). The supernatant was collected and transferred into autosampler glass vials, which were stored at −80°C until further analysis.

FRCs, B16.F10, or 4T1 (7.5 × 10^4^) were seeded onto six-well plates and grown for 24 hours in full growth medium. Medium was then replaced by RPMI without FCS. Control (no cells) and cell-conditioned medium (200 µL) was collected from each well after 24 hours. The collected media was centrifuged for 10 minutes at 16,500 × g and 4°C and 50-µL of the supernatant was extracted in 750 µL of cold metabolite extraction solution. Following centrifugation for 10 minutes at 16,500 × g and 4°C, the supernatant was transferred onto autosampler glass vials and stored at −80°C until further analysis. Cell-conditioned medium extracts from five independent cell cultures were analyzed for each condition, as well as control (nonconditioned) cell culture medium extracts. Samples were randomized in order to avoid bias due to machine drift and processed blind.

For intracellular metabolite profiling, 5 × 10^3^ or 12 × 10^3^ FRCs were seeded onto 6six-well plates or T25 flasks respectively, treated for 4 days as stated above (Cell culture) and harvested using cell scrapers. Cells were then processed for LC–MS as frozen pellets or by adding 500 µL extraction solution as described above.

LC–MS analysis was performed using a QExactive mass spectrometer coupled to a Dionex U3000 UHPLC system (Thermo Fisher Scientific). The liquid chromatography system was fitted with a Sequant ZIC-pHILIC column (150 mm × 2.1 mm) and guard column (20 mm × 2.1 mm) from Merck Millipore and temperature maintained at 45°C. The mobile phase was composed of 20 mmol/L ammonium carbonate and 0.1% ammonium hydroxide in water (solvent A), and acetonitrile (solvent B). The flow rate was set at 200 µL per minute with the gradient described previously (45). The mass spectrometer was operated in full MS and polarity switching mode. The acquired spectra were analysed using XCalibur Qual Browser and XCalibur Quan Browser software (Thermo Fisher Scientific). Absolute quantification of metabolites in the cell-culture medium and lymph nodes was performed by interpolation of the corresponding standard curves obtained from commercially available compounds running with the same batch of samples.

For isotopic flux analysis of metabolites within lymph nodes, LNs were extracted and immediately homogenized in 19*vol. water in Eppendorf tubes using a potter elvehjem homogenisator equipped with a stainless steel pistil (10 strokes at 1,200 rpm). For metabolite analysis, 50 µL of the resulting homogenate were diluted with 30 µL 0.1 M HCl. Cell pellets were mixed with 76.5 µL 0.1 M HCl. For metabolite extraction, 190 µL 0.01 µmol/L lamivudine (Sigma-Aldrich, #PHR1365) and 0.5 µmol/L sucrose (Sigma-Aldrich, #1.07687) in MeOH, 20 µL 0.1 mmol/L each of ibuprofen (Sigma-Aldrich, #PHR1004), LPA-(17:0; Sigma-Aldrich, #857127P), LPC-(17:0; Sigma-Aldrich, #855676C), D7-cholesterol (Sigma-Aldrich, #700041P), and D15-octanoic acid (Sigma-Aldrich, #448214) in CHCl_3_ (Sigma-Aldrich, #1024471000)/MeOH (50/50, v/v) were added to the samples, mixed, and treated with ultrasound (5 x 1 second 250 W; Branson Ultrasonics 250 equipped with a 13-mm Disintegrator-Sonotrode (Thermo Fisher Scientific). After addition of 100 µL water and 90 µL CHCl_3_, the sample was mixed again and 100 µL CHCl_3_ was added. Samples were mixed vigorously and centrifuged in an Eppendorf centrifuge (2 minutes at 20100 rcf). Phases were separated into new Eppendorf tubes and reextracted with 150 µL of the corresponding synthetical lower [MeOH/CHCl_3_/water (70/40/10, v/v/v)] and upper phase [MeOH/CHCl_3_/water (5/58.3/60, v/v/v)] respectively. Combined upper phases were evaporated for 15 minutes at 45°C under a stream of nitrogen gas and further evaporated to dryness in a centrifugal evaporator. Combined lower phases were evaporated to dryness at 45°C under a stream of nitrogen gas. Upper phase residues (water-soluble metabolites) were dissolved in 50 µL, 5 mmol/L NH_4_OAc (Sigma-Aldrich, #73594) in CH_3_CN (Sigma-Aldrich, #1000292500)/water (50/50, v/v) prior to LC–MS analysis using a QExactive mass spectrometer coupled to a Dionex U3000 UHPLC system (Thermo Fisher Scientific). For analysis of water-soluble metabolites, the liquid chromatography system was fitted with an Acclaim Mixed-Mode HILIC-1 column (150 mm × 2.1 mm) from Thermo Fisher Scientific. The mobile phase was composed of 5 mmol/L NH_4_OAc in CH_3_CN/water (40/60, v/v; solvent A), and 5 mmol/L NH_4_OAc in CH_3_CN/water (95/5, v/v; solvent B). The column temperature was maintained at 30°C and the flow rate was set at 200 µL per minutes. For metabolite elution, the following gradient was applied: 100% solvent B for 2 minutes, linear decrease to 10% solvent B over 23 minutes, maintenance at 10% solvent B for 16 minutes, and increase to 100% solvent B over 2 minutes. The column was maintained at 100% solvent B for 7 minutes for column equilibration before each injection. The eluent was directed to the electrospray ionization source of the Q-Exactive mass spectrometry from 2.1 minutes to 38.0 minutes after sample injection. The mass spectrometer was operated in full MS and polarity switching mode applying the following MS parameters: scan range, 69 to 1,000 m/z; resolution, 70,000; automatic gain control-target, 3E6; maximum injection time, 200 milliseconds; sheath gas, 30; auxiliary gas, 10; sweep gas, 3; spray voltage, 3.6 kV (positive mode) or 2.5 kV (negative mode); capillary temperature, 320°C; S-lens RF level, 55.0; and auxiliary gas heater temperature, 120°C. Annotation and data evaluation: peaks corresponding to the calculated monoisotopic masses (MIM ± H+ ± 2 mMU) were integrated using TraceFinder software (Thermo Fisher Scientific, version 3.3.350.0).

Metabolite levels were normalized on external standards, cell number, protein content, or total metabolite area.

### Cytokine array

Cytokine arrays were performed according to the R&D System Proteome Profiler Mouse XL Cytokine Array Kit (R&D Systems, #ARY028) product manual. Eight milliliters of medium from 3 × 10^6^ B16.F10 tumor cells cultured in a T75 culture flask was collected containing 2% FBS, 24 hours after seeding and centrifuged at 300 × g to remove cell debris. For analysis of tumor lysates, tumors were excised from mice, homogenized in RIPA buffer using a tissue grinder, and subsequently spun at 16,250 × g for 10 minutes. A bicinchoninic acid (BCA; Thermo Fisher Scientific) assay of the supernatant was performed to quantify protein content and 1 mg of tumor lysate and 1 mL of conditioned medium was used per membrane of the cytokine array. Analysis was done by use of ImageJ with the “Protein Array Analyser” Macro.

### Statistical analyses

Statistical analyses were performed using GraphPad Prism 8 software (GraphPad). For comparisons of three or more groups, data were subjected to one-way or two-way ANOVA analysis, followed by posthoc test (Dunnett when comparing every mean with a control mean, or Tukey multiple comparisons test when comparing every mean to every other mean). When two groups were compared, a two-tailed unpaired Student *t* test was applied. Data are represented as mean ± SD or SEM, and *P* ≤ 0.05 was considered significant.

### Generation and analysis of the computational model

The computational model was developed in the BioModelAnalyzer (BMA; http://biomodelanalyzer.org). The BMA allows generation of complex, discrete, executable models using a simple user interface, before models can be interrogated through simulation analysis, stability analysis, and linear temporal logic. Nodes in the model are generally defined as representing a single protein, gene, or metabolite species, and changes in the discrete value of this node represent alterations in the concentration or activity of that species. In this model, most nodes have a range of 0 to 4, where 2 is defined as normal activity/concentration, 0 to 1 as low activity/concentration, and 3 to 4 as high activity/concentration. Where nodes differ from this they have been annotated in the model file, a notable example is the OCR node, which ranges from 0 to 10, from low to high activity respectively. This change was made to more accurately compare values to experiments.

Nodes in the BMA are linked through simple mathematical operations that describe how two nodes relate to each other (called a target function). A target function contains a simple mathematical operation, the result of which describes the value at which a node will tend to. At each step of a simulation of proof analysis, a node may change by a single value (up by one, down by one, or no change), and will tend towards the value of its target function. Nodes are updated in synchrony each step of a simulation. Target functions can encapsulate inputs from multiple sources, and can generate extremely complex regulatory relationships.

The core metabolic network is generally well understood, and as such was generated primarily from literature (46). Metabolic analysis was performed with the use of a special series of nodes. We included a “counter” node, which increases over the course of a simulation in which a metabolic analysis experiment is run from 0 to 100, roughly the number of minutes of a standard metabolic analysis experiment. When the “counter” node reaches values equivalent to the timings of drug applications, the model induces a change in the activity of nodes equivalent to the application of the drug, in this way we are able to apply different drug treatments to the model at set intervals, replicating the temporal properties of the experiments. All models included in this manuscript are available at https://github.com/shorthouse-mrc/biomodelanalyzer_lactate, and can be run by loading them into the BioModelAnalyzer (http://biomodelanalyzer.org).

### Data availability

The data generated in this study are publicly available in the Gene Expression Omnibus (GEO) database (GEO number GSE184300).

## Results

### Soluble factors mediate lymph-node expansion and reprogramming

**Figure 1.**
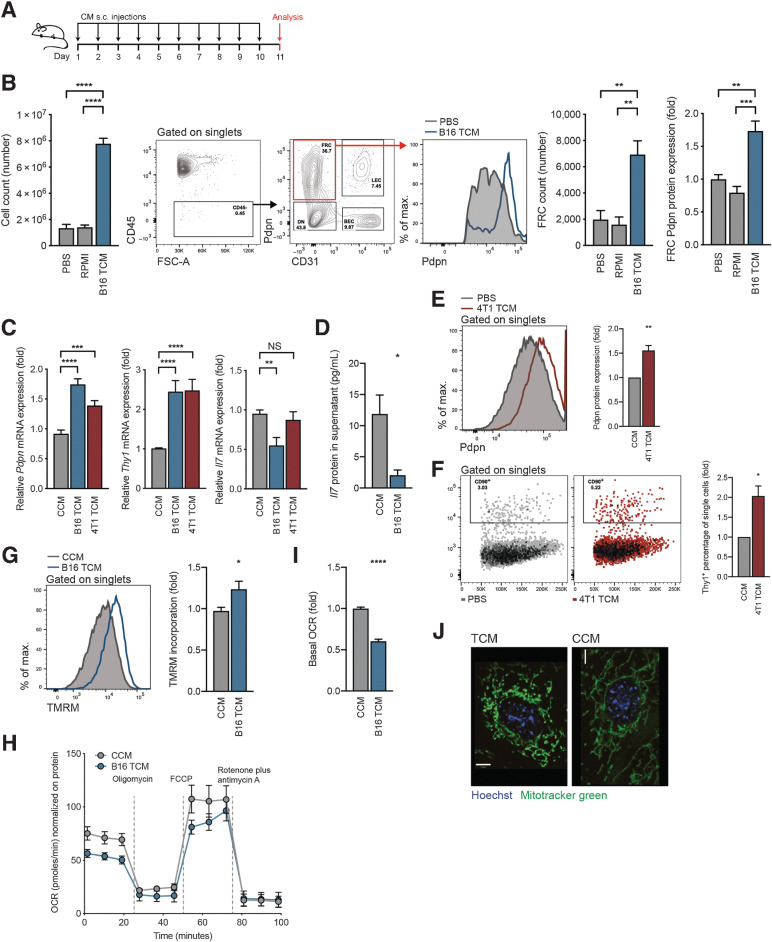
Soluble factors mediate FRC activation and mitochondrial imbalance. **A,** Experimental scheme used to investigate tumor-draining factors *in vivo*. **B,** Draining lymph nodes were harvested from female C57BL/6 mice that had received 10 daily subcutaneous doses of PBS, RPMI medium, or B16.F10 TCM into the shoulder. Flow cytometric analysis allowed reporting of the total number of lymph node cells (left), flow cytometry gating scheme, FRCs (right), and FRC Pdpn histogram (furthest right). *n* = 4 to 9 animals per group. **C,** qRT-PCR analysis of *Pdpn* (left), *Thy1* (middle), and *Il7* (right) in FRCs cultured *in vitro* and treated for 4 days with CCM, B16.F10 TCM, or 4T1 TCM. *n* = 3 to 10 independent experiments. **D,** IL7 protein quantification in supernatants of FRCs cultured *in vitro* and treated with CCM or B16.F10 TCM, assessed by ELISA. *n* = 4 independent experiments. Flow cytometric quantification of Pdpn protein **(E)** and Thy1 protein **(F)** expression for FRCs cultured *in vitro* and treated with CCM or 4T1 TCM for 4 days. *n* = 3 independent experiments. **G,** Flow cytometric analysis of TMRM incorporation by FRCs treated for 4 days with CCM or B16 TCM. *n* = 4 independent experiments performed in triplicate. **H,** OCR of FRCs treated with CCM or B16.F10 TCM at baseline and in response to oligomycin, FCCP, and rotenone plus antimycin A. One representative of three experiments with five replicates. **I,** Baseline OCR from CCM or B16.F10 TCM-treated FRCs. *n* = 3 experiments with 5 replicates. **J,** Representative confocal imaging of live cells treated with CCM or B16.F10 TCM. Green, Mitochondria (Mitotracker green); blue, nuclei (Hoechst). Scale bar: 7 µm. Data are mean with SEM. Significance (*, *P* < 0.05; **, *P* < 0.01; ***, *P* < 0.001; and ****, *P* < 0.0001) was determined by unpaired two-tailed *t* test or one-way ANOVA with Tukey *post hoc* (**B**). s.c., subcutaneous; B16, B16.F10; max, maximum; NS, not significant.

To determine the underlying drivers of FRC reprogramming, we first tested whether the expansion and reprogramming of stroma in TDLNs is mediated by either physical cues, increased drainage, or draining soluble factors. To this end, in the absence of tumor cells, C57BL/6 mice received daily subcutaneous doses of PBS, RPMI medium, or B16.F10 TCM into the shoulder for a period of 11 days (Fig. 1A). TCM was sufficient to induce increased total lymph node cellularity, FRC numbers, and Pdpn expression by FRCs (Fig. 1B). Neither PBS nor RPMI media impacted the lymph node or FRCs, indicating that physical cues from elevated fluid drainage alone were not driving the observed changes. Pdpn and Thy1 are commonly used as CAF markers (47–49) and their upregulation in TDLNs is thought to play a role in establishing the premetastatic niche. We previously showed that both are upregulated in FRCs of melanoma TDLNs and that IL7, a lymph node–critical cytokine is downregulated (39). This was confirmed here for *Thy1* and *Il7* in 4T1 breast cancer TDLNs (Supplementary Fig. S1A). *In vitro*, FRCs stimulated with B16.F10 or 4T1 TCM significantly upregulated *Pdpn* and *Thy1* and downregulated *Il7* compared with FRCs stimulated with CCM (Fig. 1C), mirroring the *in vivo* observations. This was confirmed at the protein level for IL7 in FRCs stimulated with B16.F10 TCM (Fig. 1D) and for Pdpn and Thy1 in FRCs stimulated with 4T1 TCM (Fig. 1E and F).

In our previous study, oxidative phosphorylation and mitochondrial dysfunction gene signatures were perturbed in TDLN FRCs (39). Thus, we investigated whether TCM exerted a similar impact. When compared with CCM-treated FRCs, those treated with B16.F10 TCM exhibited increased incorporation of TMRM, a cell-permeable mitochondrial membrane dye whose accumulation is proportional to mitochondrial potential (Fig. 1G). Furthermore, in a mitochondrial stress test, both basal and maximal OCR were reduced in FRCs stimulated with B16.F10 TCM (Fig. 1H and I). These changes led us to investigate mitochondrial morphology, which is a readout of mitochondrial function. Mitotracker green staining of live cells showed that mitochondria of CCM-treated FRCs were elongated and tubular in appearance. In contrast, B16.F10 TCM–treatment induced a punctate phenotype mitochondria, further indicating alterations in mitochondrial function (Fig. 1J).

These data suggested that soluble, tumor-derived factors were mediating lymph-node expansion and FRC reprogramming. With lymph containing an array of components, including microvesicles, proteins, soluble antigen, nucleic acids, and metabolites, linked to formation of premetastatic niches, we sought to further delineate the factor in question. Analysis of FRCs treated *in vitro* with either <3kDa or >3kDa B16.F10 TCM confirmed that upregulation of *Pdpn* and *Thy1* mRNA was mediated by small molecular weight factors below 3kDa (Supplementary Fig. S1B). Tumor-derived exosomes had no impact on *Pdpn* expression, but a strong induction was retained by exosome-depleted supernatant (Supplementary Fig. S1C). To further corroborate that effects were mediated by tumor-secreted factors below <3kDa, we performed a cytokine array (Supplementary Fig. S1D) and then stimulated FRCs with the two most significantly expressed proteins, angiopoeitin-2 and osteopontin. These cytokines had no impact on either *Pdpn*, *Thy1*, or *Il7* mRNA expression (Supplementary Fig. S1E), nor did treatment with TGFβ (Supplementary Fig. S1F). Further depletion approaches using freeze/thawing, boiling and DNase treatment had little effect on *Pdpn* and *Thy1* mRNA levels (Supplementary Fig. S1G), indicating that the factors responsible for changes in expression of these markers were likely small molecule metabolites.

### Lactate induces mitochondrial and activation reprogramming in lymph node FRCs

**Figure 2.**
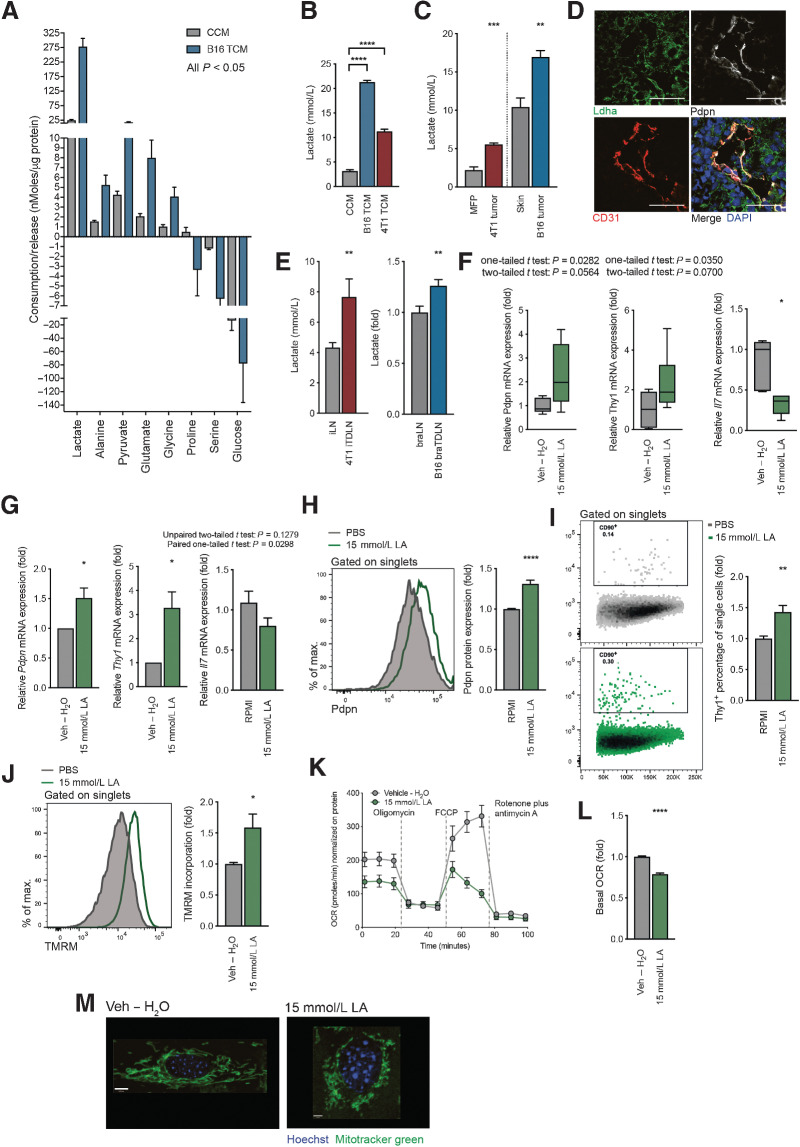
Tumor-derived lactate is responsible for the observed changes in FRC activation and mitochondria. **A,** Metabolomic analysis of amino acids and glycolysis- and TCA cycle–intermediates in CCM or B16.F10 TCM using LC–MS. Metabolites with a significant (*P* < 0.05) FC are shown, ordered according to nMoles/µg protein of cultured cells. *n* = 5 independent experiments. **B,** L-lactate concentration in CCM, B16.F10 TCM, and 4T1 TCM, as measured by an enzymatic assay. *n* = 3 experiments with 1 to 5 replicates. **C,** L-lactate quantification in tissues collected from either Balb/c sham-treated mice (MFP) and orthotopic 4T1 tumors (4T1 tumor), or C57BL/6 sham-treated (skin) and B16.F10 melanoma tumors (B16 tumor). Measured by an enzymatic assay. *n* = 4 to 6 independent experiments. **D,** Confocal image of a B16.F10 tumor. Lymphatic vessels are detected with Pdpn (white) and CD31 (red). Ldha-positive tumor cells (green) and nuclei (blue). Scale bar: 100 µm. Representative of 3 experiments with 3 views each. **E,** Quantification of L-lactate concentration in lymph nodes from mice in (**C**).Inguinal lymph nodes (iLN) were harvested from sham-treated and orthotopic 4T1 tumor-bearing (4T1 iTDLN) Balb/c mice and L-lactate measured by an enzymatic assay. *n* = 2 to 7 biological replicates. Brachial lymph nodes (braLNs) were harvested from sham-treated and B16.F10 tumor-bearing C57BL/6 mice (B16 braTDLNs). L-lactate is shown as FC quantified by either the enzymatic assay or LC–MS. *n* = 17 biological replicates. **F,** Quantitation of *Pdpn* (left), *Thy1* (middle), and *Il7* (right) mRNA expression in FRCs of draining lymph nodes from female C57BL/6 mice receiving vehicle (Veh – H_2_O) or 15 mmol/L LA s.c. daily for 11 days. FRCs were FACS sorted as Pdpn^+^CD31^–^CD45^–^. Box and whiskers plot with whiskers minimum to maximum (line displays the median). Mean with SD. *n* = 4 animals with two lymph nodes for each experiment. **G,** Quantification of *Pdpn* (left), *Thy1* (middle), and *Il7* (right) mRNA in FRCs treated with vehicle (Veh – H_2_O) or 15 mmol/L LA for 4 days *in vitro*. *n* = 4 to 6 experiments. Flow cytometric quantification of Pdpn (**H**) and Thy1 protein expression (**I**), and TMRM incorporation (**J**) by FRCs treated with Veh – H_2_O or 15 mmol/L LA for 4 days *in vitro*. *n* = 5 experiments in triplicate (**H**). *n* = 3 experiments in triplicate (**I**). *n* = 4 experiments in duplicate (**J**). **K,** OCR of FRCs treated with vehicle (Veh – H_2_O) or 15 mmol/L LA at baseline and in response to oligomycin, FCCP, and rotenone plus antimycin A. Representative data of 3 experiments with 5 replicates. **L,** Baseline OCR from vehicle (Veh – H_2_O) and 15 mmol/L LA–treated FRCs. *n* = 4 experiments with 5 replicates. **M,** Representative confocal images of live cells treated with vehicle (Veh – H_2_O) or 15 mmol/L LA. Green, Mitochondria (Mitotracker green); blue, nuclei (Hoechst). Scale bars: 8 µm (vehicle), 3.3 µm (15 mmol/L LA). Representative of three independent experiments. Data are means with SEM. Significance (*, *P* < 0.05; **, *P* < 0.01; ***, *P* < 0.001; and ****, *P* < 0.0001) was determined by unpaired two-tailed *t* test or one-way ANOVA with Tukey *post hoc* (**B**). B16, B16.F10; max., maximum; min, minute; LA, lactic acid.

To narrow-down factors below 3kDa driving the observed functional changes in FRCs, we performed metabolomics analysis of CCM and B16.F10 TCM using LC–MS (Fig. 2A). Although additional metabolites may contribute, lactate and pyruvate were most enriched in B16.F10 TCM, consistent with B16.F10 cells having a glycolytic phenotype. We focused on lactate, which showed more than 10-fold higher levels than any other metabolite, confirming that both B16.F10 and 4T1 TCM had increased levels of lactate in an enzyme-based assay compared with CCM (Fig. 2B). We then quantified levels in tumors, finding that lactate was enriched in 4T1 orthotopic breast tumors compared with mammary fat pad, and in B16.F10 orthotopic melanomas compared with skin (Fig. 2C). Within B16.F10 tumors, LDHA, the enzyme that converts pyruvate to lactate, was detected in close proximity to lymphatic vessels, supporting the hypothesis that tumor-derived lactate drains to TDLNs where it may influence FRC behavior (Fig. 2D). Further evidence to support this was provided by the observation of higher lactate concentrations in TDLNs than in sham control lymph nodes for both tumor models (Fig. 2E).

To account for the difficulties in capturing metabolites and metabolite fluxes draining from a tumor at distant sites *in vivo*, we subcutaneously injected TCM from ^13^C-glucose–labeled 4T1 tumor cells (4T1 TCM) daily for 11 days at the shoulder in lieu of a tumor. This site drains specifically to the brachial lymph node, where labeled metabolites including glucose, pyruvate, lactate, glycerol-3-P, and adenosine were detected (Supplementary Fig. S1H). Next, we subcutaneously injected 15 mmol/L LA daily into the shoulder. This approach confirmed that draining lymph nodes reacted to LA via a slight increase in cell count (Supplementary Fig. S1I), by upregulation of *Pdpn* and *Thy1*, and by downregulation of *Il7* in FRCs (Fig. 2F). Moreover, FRCs exposed to 15 mmol/L LA *in vitro*, also displayed significant upregulation of both *Pdpn* and *Thy1* and a slight downregulation of *Il7* (Fig. 2G), whereas changes induced by low pH medium (RPMI pH6) and 15 mmol/L sodium lactate, a conjugated base of LA, were less potent (Supplementary Fig. S1J). Pdpn and Thy1 upregulation was also verified at the protein level with 15 mmol/L LA treatment (Fig. 2H and I). Interfering with Ldha/Ldhb by using an inhibitor (GSK 2837808A) decreased secretion of LA by cultured B16.F10 tumor cells (Supplementary Fig. S1K). Using LA–low TCM, we observed a significant reduction in the effect on *Thy1* mRNA levels, whereas *Pdpn* mRNA levels were only slightly affected (Supplementary Fig. S1 L and S1M).

As with TCM, LA treatment led to increased TMRM accumulation (Fig. 2J) in FRC mitochondria, and mitochondrial stress tests (Fig. 2K) revealed functional differences, especially in the basal OCR (Fig. 2L), whereas sodium lactate and low pH medium did not (Supplementary Fig. S2A and S2B). Moreover, a switch in mitochondrial phenotype mirroring those of TCM treatment were detected following LA exposure; elongated mitochondria with vehicle treatment versus induction of punctated mitochondria with sodium lactate treatment (Fig. 2M). Coincident with this phenotypic change, an increase in MTG incorporation was also measured following LA treatment, which is indicative of mitochondrial mass alterations (Supplementary Fig. S2C). Of note, LA and TCM did not affect viability (Supplementary Fig. S2D) and proliferation (Ki67 staining, Supplementary Fig. S2E), consistent with previous studies (50). In summary, these data indicate that tumor-derived LA is sufficient to recapitulate mitochondrial changes and activation signatures induced by TCM in FRCs of TDLNs.

### Lactic acid induces lymph node FRC extracellular matrix and immune signatures

**Figure 3.**
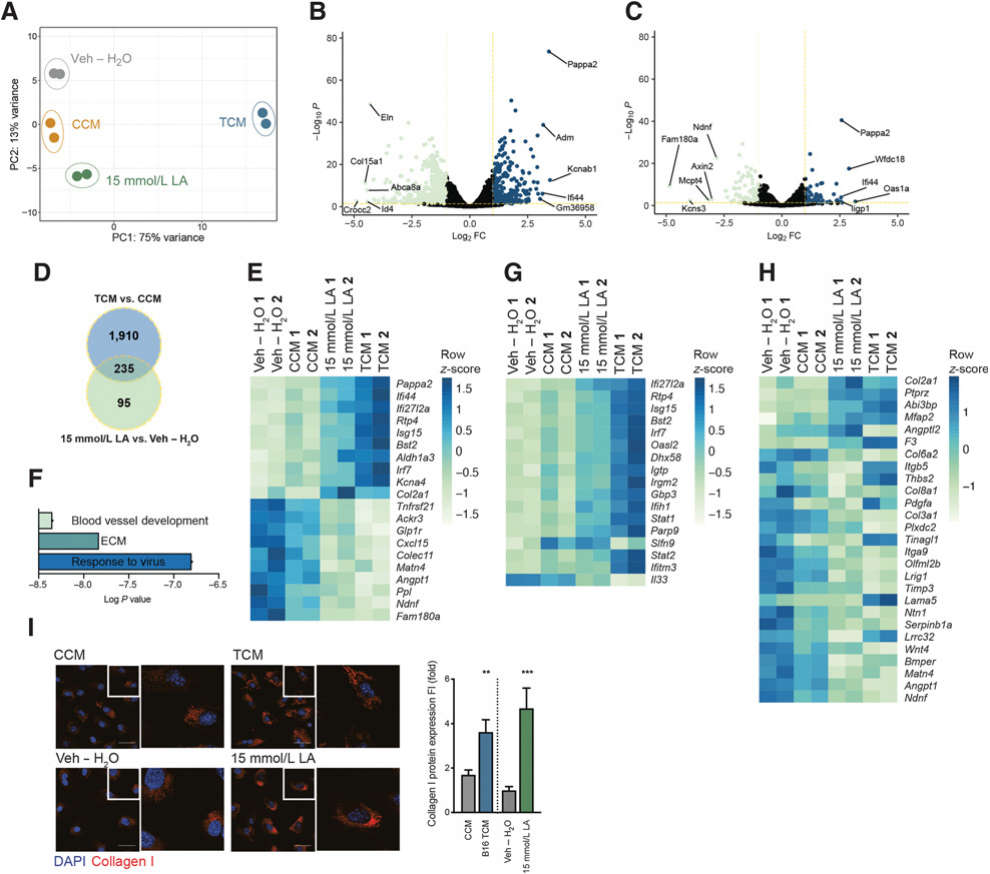
Tumor factors and LA induce FRC remodeling. **A,** PCA plot of RNA-seq data calculated for the 500 genes showing the highest variance in a variance stabilizing transformed matrix with log_2_-transformed data from *in vitro* FRCs treated with CCM, B16.F10 TCM, vehicle (Veh – H_2_O) or 15 mmol/L LA for 4 days. *n* = 2 biological replicates. **B,** Volcano plot comparing genes expressed by *in vitro* cultured FRCs treated for 4 days with TCM or CCM, the *x*-axis displays the significance (-log_10_*P*) and the *y*-axis displays the log_2_ FC. Yellow lines are at -log_10_*P* = 1.3 and log_2_ FC 0.68. The top and bottom five are labeled. **C,** Volcano plot comparing genes expressed by FRCs treated with 15 mmol/L LA or Veh – H_2_O for 4 days *in vitro*; plot labels as in (**B**). **D,** Overlap of genes significantly deregulated in FRCs treated with TCM versus CCM and 15 mmol/L LA versus Veh – H_2_O for 4 days *in vitro*. **E,** Heatmap displaying the top and bottom 10 most deregulated genes of the 235 genes overlapping in **D**. **F,** Summary of key pathways identified in *in vitro* cultured FRCs treated for 4 days with 15 mmol/L LA versus Veh – H_2_O using genes with -log_10_*P* < 1.3 and log_2_ FC > 0.68. Detailed analysis in Supplementary Fig. S2. **G,** Heatmap displaying significant deregulated genes in 15 mmol/L LA versus Veh – H_2_O (including data for TCM vs. CCM) within the ”response to virus”/IFN signature. **H,** Heatmap displaying significant deregulated genes in 15 mmol/L LA verus Veh – H_2_O (including data for TCM vs. CCM) within the ”ECM” signature. **I,** Confocal images of FRCs treated for 4 days with CCM, B16.F10 TCM, Veh – H_2_O, or 15 mmol/L LA and stained for collagen I (red) and nuclei (blue; left) and quantification thereof (right). *n* = 2 independent experiments each with 8 fields of view analyzed. Scale bar: 50 µm. Data are mean with SEM. Significance (*, *P* < 0.05; **, *P* < 0.01; ***, *P* < 0.001; and ****, *P* < 0.0001) was determined by unpaired two-tailed *t* test. B16, B16.F10.

To identify FRCs components deregulated in response to B16.F10 TCM and LA exposure, we analyzed changes in the transcriptome. Applying principle component analysis (PCA; Fig. 3A) based on differentially regulated genes, we observed a gradual shift in gene expression patterns from vehicle (H_2_O), CCM, 15 mmol/L LA through to TCM-treated FRCs. Two thousand one hundred and forty-five genes were significantly differentially expressed between TCM and CCM treatment (Fig. 3B) and 330 genes differed when comparing 15 mmol/L LA and vehicle (H_2_O) treatment (Fig. 3C). Of these, 235 were overlapping (Fig. 3D and E). Gene ontology (GO) enrichment analysis (biological process and cellular component) revealed that significantly differentially regulated genes, −log_10_*P* < 1.3 and log_2_ FC > 0.68, between 15 mmol/L LA and vehicle treatment were mainly involved in processes related to response to virus (IFN response), extracellular matrix (ECM), and blood vessel development (Fig. 3F; Supplementary Fig. S2F and S2G). Most of the genes deregulated by 15 mmol/L LA falling into the category ”response to virus” were also deregulated by TCM treatment (Fig. 3G). Similarly, LA–induced deregulation of ECM-related genes was recapitulated by TCM, with additional gene adaptations that likely reflect the additional complexity of TCM composition (Fig. 3H). Moreover, both the ECM and the interferon signatures were detected in transcriptomic profiles of sorted FRCs from TDLNs (Supplementary Fig. S2H and S2I ref. 39). Confocal imaging confirmed a significant increase in collagen1 at the protein level in both 15-mmol/L LA– and TCM-treated FRCs (Fig. 3I). A TF analysis implementing the deregulated genes after 15-mmol/L LA stimulation revealed the involvement of many embryonic TFs, such as Sox2, Nanog, Pouf5f1, and Oct4, as well as TFs linked to metabolism, such as Ppard, which is critical in fatty acid metabolism (Supplementary Fig. S2J). PCR arrays further confirmed deregulated genes associated with fibrosis in FRCs treated with TCM and 15 mmol/L LA *in vitro* (Supplementary Fig. S2K–S2N).

### Lymph node FRCs modify their metabolism in response to tumor-derived factors or lactic acid

**Figure 4.**
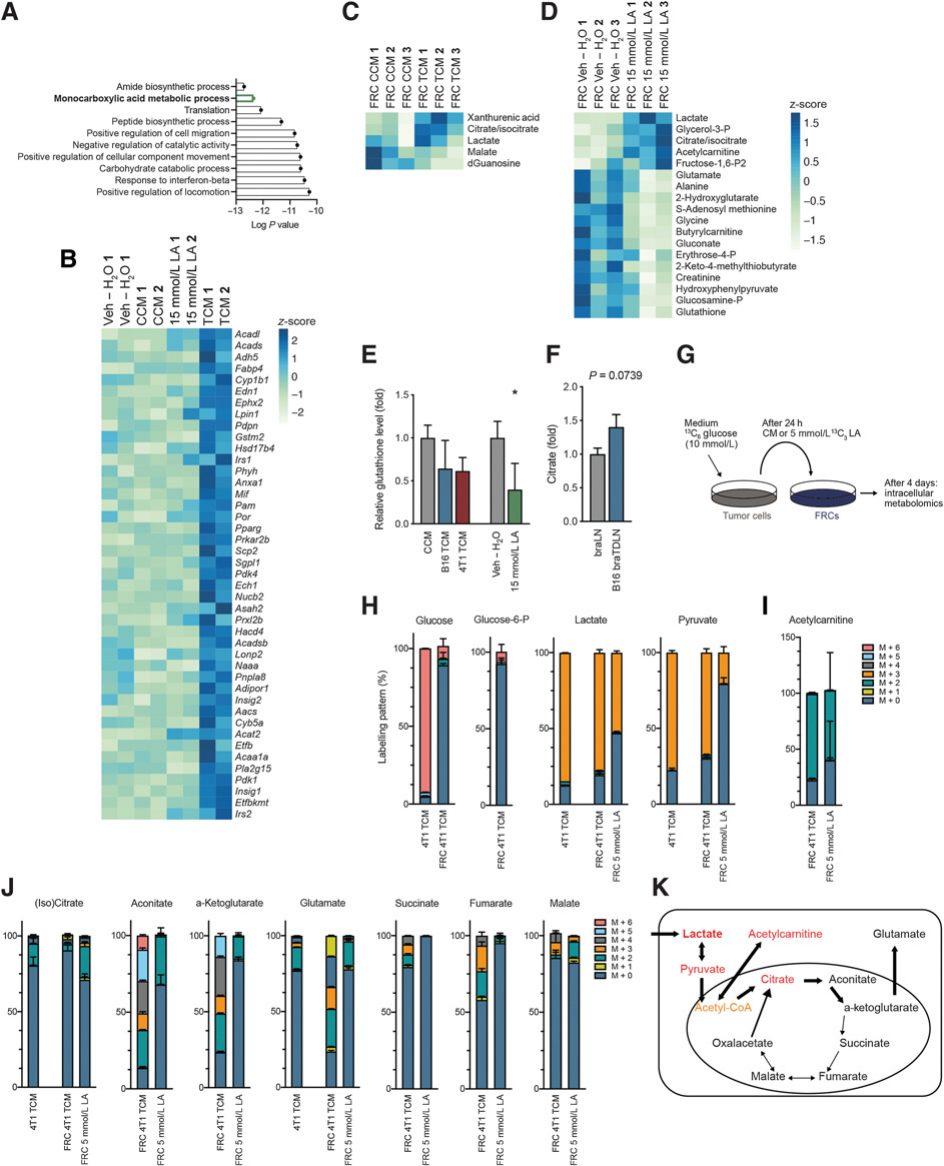
Deregulated metabolism in response to tumor factors and LA. **A,** Using the RNA-seq data from Fig. 3, depicted here are the top 10 most significant GO terms for ”biological process” using all significantly upregulated genes in *in vitro* cultured FRCs treated for 4 days with B16.F10 TCM versus CCM. **B,** Heatmap displaying significant deregulated genes in TCM versus CCM (including data for 15 mmol/L LA vs. Veh – H_2_O) within ”fatty acid metabolism”. **C,** Significant intracellular (*P* < 0.05) metabolites in FRCs treated for 4 days with B16.F10 TCM versus CCM *in vitro*, measured by LC–MS and normalized on protein level. **D,** Significant intracellular (*P* < 0.05) metabolites in FRCs treated for 4 days with 15 mmol/L LA versus Veh – H_2_O *in vitro*, measured by LC–MS and normalized on cell number. **E,** Relative intracellular glutathione levels in FRCs treated for 4 days with CCM, B16.F10 TCM, 4T1 TCM, 15 mmol/L LA, or Veh – H_2_O *in vitro*, measured by LC–MS. *n* = 3 independent experiments. **F,** Relative abundance of citrate in brachial lymph nodes of female sham-treated (braLNs) and B16.F10 tumor-bearing C57BL/6 mice (B16 braTDLN), quantified by LC–MS. *n* = 7 independent experiments.**G,** Experimental set-up for labeling experiment. 4T1 tumor cells were cultured in ^13^C_6_-glucose medium for 24 hours before medium was transferred to FRCs. In parallel, FRCs were cultured with ^13^C_3_ LA directly. In both cases, FRCs were treated for 4 days and metabolites measured by LC–MS. **H–J,**^13^C-labeled metabolites identified by LC–MS using the set-up in **G**. **H,** glucose, glucose-6-P, lactate, and pyruvate. **I,** acetylcarnitine and **J,** citrate, aconitate, a-ketoglutarate, glutamate, succinate, fumarate, and malate. All *n* = 3 technical replicates. “M + n”, molecular mass plus the number of incorporated heavy carbons. Data are mean with SD. **K,** Diagram showing incorporation of lactate and pyruvate into downstream metabolites. Data are mean with SEM (unless stated differently). Significance (*, *P* < 0.05; **, *P* < 0.01; ***, *P* < 0.001; and ****, *P* < 0.0001) was determined by unpaired two-tailed *t* test. B16, B16.F10; h, hours; LA, lactic acid.

To determine how LA impacts FRC molecular and functional metabolic changes, we performed GO analyses on the significantly differentially expressed genes from the RNA-seq data, comparing B16.F10 TCM with CCM. A significant upregulation of genes required for monocarboxylic acid metabolism (Fig. 4A), which predominantly fell into the category of lipid/fatty-acid oxidation (Fig. 4B), indicated a shift in intracellular metabolism. Metabolic profiling of 200 water-soluble intracellular metabolites *in vitro* identified five significantly deregulated metabolites in TCM-treated FRCs compared with CCM (Fig. 4C), and 19 significantly deregulated metabolites in FRCs treated with 15 mmol/L LA compared with vehicle (H_2_O; Fig. 4D). This included an increase of intracellular lactate and citrate/isocitrate in both conditions. With the exception of lactate, changes in these metabolites were not detected in their respective media (Supplementary Fig. S3A), indicating that rather than being taken up from the media, they were synthesized by FRCs in response to TCM or LA. This analysis further confirmed that of the metabolites examined, only lactate was significantly enriched in TCM (Supplementary Fig. S3B). Many metabolites that decreased in FRCs treated with 15 mmol/L LA were involved in glutathione formation, including glutamate, glycine, creatinine, and glutathione, which indicates a possible reduction in antioxidant capacity. Intracellular glutathione levels in FRCs were also reduced, albeit not significantly, in response to TCM from B16.F10 or 4T1 cells (Fig. 4E). Similarly, both TCM and LA treatment *in vitro*, elevated intracellular citrate/isocitrate levels in FRCs compared with respective CCM and vehicle (H_2_O) controls (Fig. 4C and D). Trending increases of citrate/isocitrate were also detected in TDLNs *in vivo* (Fig. 4F), indicating its synthesis *in vivo* in response to tumor factors.

We used ^13^C-labeled glucose in the medium of 4T1 breast cancer cells to label tumor-derived metabolites. After 24 hours of culture, this tumor-metabolite labeled conditioned medium (CM) was used to treat FRCs. Concurrently, FRCs were directly labeled using 5 mmol/L ^13^C_3_ LA. After 4 days of culture, labeled metabolites were measured intracellularly (Fig. 4G). The 4T1 TCM generated was also subjected to metabolomic analysis. Although 4T1 TCM almost exclusively contained labeled glucose, glucose and glucose-6-P within FRCs treated with 4T1 TCM (FRC 4T1 TCM) was almost entirely unlabeled (Fig. 4H). Lactate and pyruvate were heavily labeled in 4T1 TCM, and also within FRCs treated with 4T1 TCM or LA (Fig. 4H), suggesting lactate uptake and subsequent conversion to pyruvate. Pyruvate forms acetyl-CoA, which feeds into the tricarboxylic acid (TCA) cycle. As cell numbers in our sample set were low, we experienced technical limitations in measuring acetyl-CoA. However, we could measure acetylcarnitine, which was highly enriched in LA-treated FRCs and was labeled in 4T1 TCM or ^13^C_3_ LA-treated FRCs (Fig. 4D and I). In principle, acetyl-CoA can be incorporated into the TCA cycle, or into acetylcarnitine, if levels are increased and/or the TCA cycle is inhibited. Citrate/isocitrate labeling was hardly detectable (Fig. 4J), but this could be masked by citrate/isocitrate uptake from 4T1 TCM and accumulation in the cytosol. Aconitate and α-ketoglutarate were not increased, but they and glutamate were labeled, whereas labeling of other TCA cycle intermediates, such as succinate, fumarate, and malate was barely detectable (Fig. 4J). In summary, this indicated that FRCs used lactate and pyruvate to form acetylcarnitines and α-ketoglutarate/glutamate, rather than the TCA cycle for energy production, contributing to the observed reduction of oxidative phosphorylation (Fig. 4K).

### Lymph node FRC activation and mitochondrial changes are dependent on intracellular pH

Having observed effects of tumor-derived cues, especially lactate, on FRC activation phenotype and metabolic function, we sought to determine the underlying mechanisms of action. Previous reports suggest that lactate can exert its effects on target cells such macrophages (13) and endothelial cells (28) in tumors via Hif1α. We found that although FRCs were responsive to hypoxia (1% O_2_), 15 mmol/L LA had no impact on Hif1α relocalization to the nucleus from the cytoplasm compared with vehicle controls (Supplementary Fig. S3C). Moreover, mRNA levels of the Hif1α target genes *Ldha* and *Vegfa* did not change in response to LA, but a significant downregulation of *Gapdh* was observed (Supplementary Fig. S3D). Together these results implied that Hif1α-driven signaling pathways are not required for LA–driven FRC reprogramming in TDLNs. Given that tumor-derived LA impacts several pathways in FRCs, it is likely that rather than relying on the activation of a single TF, LA induces a more global effect in FRCs. To further explore this, we generated an executable model of the TCA cycle, including a function for predicting OCR *in silico*. Using the model, we simulated a metabolic stress test to examine the import of lactate and protons, and their corresponding effects on glycolysis and the TCA cycle.

**Figure 5.**
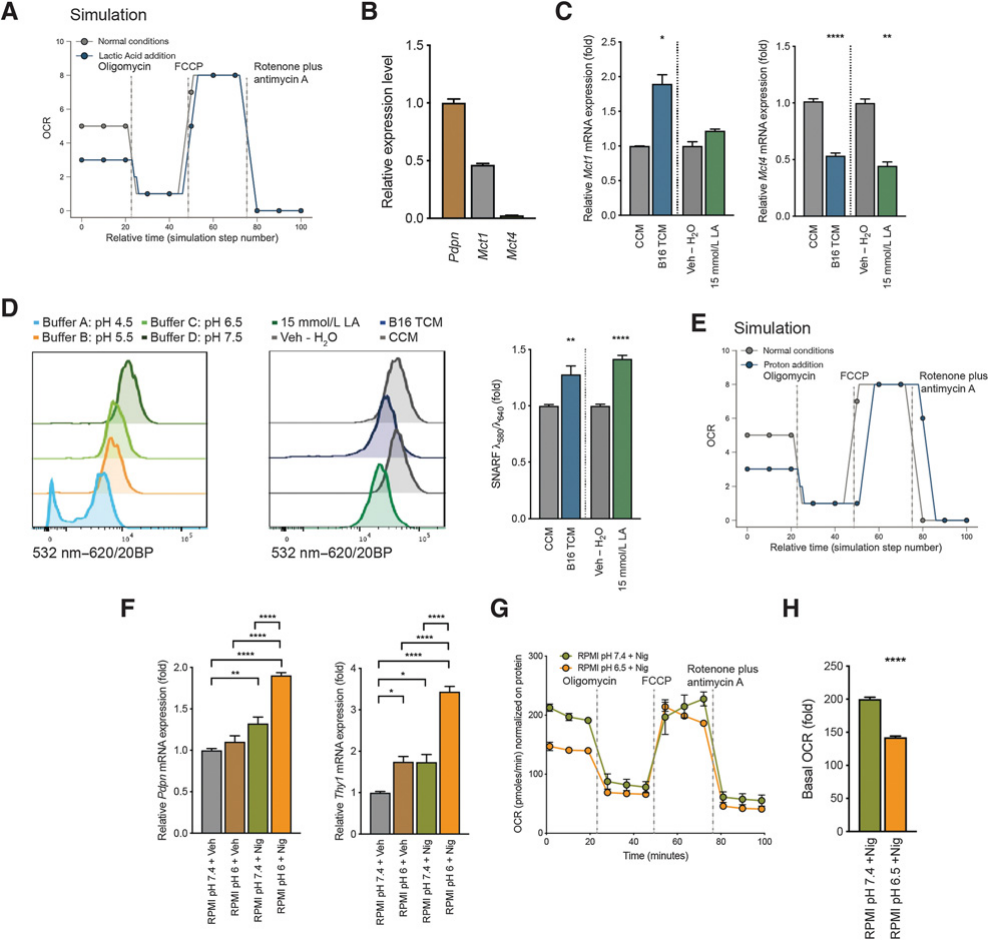
Intracellular pH shift of FRCs contributes to mitochondrial changes and the observed signature. **A,** Metabolic analysis of mitochondrial function in the computational model. Shown is OCR for mitochondria exposed to LA versus control. **B,** Quantification of *Pdpn, Mct1*, and *Mct4* mRNA in cultured FRCs. Displayed as relative gene expression with *Actb* as housekeeping and *Pdpn* as reference gene. *n* = 3 independent experiments in duplicate. **C,** Quantification of *Mct1* (left) and *Mct4* (right) mRNA in FRCs treated with CCM, B16.F10 TCM, Veh – H_2_O, or 15 mmol/L LA for 4 days. *n* = 2 to 4 independent experiments. **D,** Intracellular pH of FRCs treated as in (**C**) and stained with cell-permeant ratiometric fluorescent pH indicator (SNARF). SNARF exhibits a pH-dependent emission shift calculated by λ_586_/λ_610_. Lower intracellular pHs give higher values. Control buffers used to adjust intracellular to extracellular pH (left). Representative histogram of the 532nm to 620/20BP shift for CCM, B16,F10 TCM, Veh – H_2_O, or 15 mmol/L LA (middle). Quantification thereof (right). *n* = 3 independent experiments in duplicate. **E,** Metabolic analysis of mitochondrial function in the computational model. Shown is OCR for mitochondria exposed to low pH (proton addition) versus control. **F,** Quantification of *Pdpn* (left) and *Thy1* (right) mRNA in FRCs cultures after 48 hours of treatment with normal pH RPMI and vehicle (RPMI pH7.4 + Veh), normal pH RPMI and 100 nmol/L nigericin (RPMI pH7.4 + Nig), pH6 RPMI and vehicle (RPMI pH6 + Veh), or pH6 RPMI and 100 nmol/L nigericin (RPMI pH6 + Nig). *n* = 2 to 3 independent experiments in duplicate. **G,** OCR of FRCs treated with pH7.4 RPMI and 100 nmol/L nigericin (RPMI pH7.4 +Nig) or RPMI at pH6.5 and 100 nmol/L nigericin (RPMI pH6.5 +Nig) at baseline and in response to oligomycin, FCCP, and rotenone plus antimycin A. Representative data of three experiments each with five replicates. **H,** Baseline OCR from FRCs treated as in **G**. Data are mean with SEM (unless stated differently). Significance (*, *P* < 0.05; **, *P* < 0.01; ***, *P* < 0.001; and ****, *P* < 0.0001) was determined by unpaired two-tailed *t* test (**A–E**) and (**G**) or one-way ANOVA with Tukey *post hoc* (**F**). B16, B16.F10; LA, lactic acid; min, minutes.

During the simulation, nodes that represent either protein activity or membrane properties were inhibited in sequence, at timings utilized experimentally. ATP synthase was inhibited to simulate oligomycin addition, membrane permeability to protons was altered to simulate FCCP addition, and a node representing mitochondrial complex I through IV was inhibited to represent the addition of rotenone and antimycin A. This enabled us to run mitochondrial stress tests *in silico* to examine the effects of disruption to metabolic pathways. The model supported experimental findings that LA reduced the basal OCR significantly in FRCs (Fig. 5A).

In light of the model predictions, we investigated whether lactate is taken up by lymph node FRCs, measuring *Mct1* (uptake) and *Mct4* (secretion) mRNA levels in FRCs *in vitro*. *Mct1* exhibited higher levels of expression than *Mct4* (Fig. 5B), and the addition of B16.F10 TCM or 15 mmol/L LA led to increased *Mct1* with a concurrent downregulation of *Mct4* compared with CCM or vehicle controls (Fig. 5C). As Mct1 is a proton-coupled monocarboxylate transporter, we hypothesized that lactate must be taken up from the extracellular space together with a proton, leading to decreased intracellular pH. Thus, intracellular pH of FRCs was measured using the cell-permeant ratiometric fluorescent pH indicator SNARF. B16.F10 TCM and 15-mmol/L LA treatment induced a significant decrease in intracellular pH compared with CCM or vehicle controls, as evidenced by increased SNARF emission shifts (Fig. 5D), whereas 15 mmol/L sodium lactate or pH 6 medium (RPMI pH 6) had no or a mild effect (Supplementary Fig. S3E). Upon further interrogation, the model predicted that addition of intracellular protons alone was enough to replicate the effects of LA on mitochondria (Fig. 5E), whereas sodium lactate addition was not enough to alter the OCR (Supplementary Fig. S3F). To further validate these predictions, we examined changes induced by a sudden decrease of intracellular pH in LA–treated FRCs *in vitro*. We mimicked the LA scenario using the potassium ionophore nigericin (Nig), which is a K^+^/H^+^ exchanger and commonly used to adjust intracellular to extracellular pH. Nigericin, a microbial toxin, has been shown to induce apoptosis via Caspase-1 (51) and reported to disturb the cell's mitochondrial bioenergetics by matrix acidification leading to a reduced basal respiration (52). Thus, we used low concentrations and short treatment durations in the following combinations: normal pH media [RPMI ph7.4 + Vehicle (Veh)], low pH media [RPMI pH6 + Vehicle (Veh)], normal pH media plus nigericin (RPMI pH7.4 + Nig), or low pH media plus nigericin (RPMI pH6 + Nig). Increased *Pdpn* and *Thy*1 mRNA levels were detected with nigericin and normal pH medium. However, expression was significantly enhanced with nigericin and a low pH medium, indicating that the induction of *Pdpn* and *Thy1* is dependent on intracellular pH and associated mitochondrial functional changes (Fig. 5F). Intracellular pH was critical for proper mitochondrial function since FRCs treated with nigericin and a low pH medium presented with functional changes in the mitochondrial stress test (Fig. 5G) and decreased basal OCR (Fig. 5H). Lactate uptake is coupled to proton uptake, thereby lowering intracellular pH. Adjustment of intracellular pH alone mirrored LA–driven effects, indicating that although lactate preferentially impacts activation and mitochondrial pathways in TDLN FRCs, its effects are mediated by a general decrease in intracellular pH.

## Discussion

Lymph nodes are key sites for the initiation of antitumor immune responses, but are also commonly invaded by cancer cells. To accommodate and support development of metastases, lymph nodes adapt, establishing a premetastatic niche. We previously demonstrated that lymph nodes immediately downstream of tumors enlarge, and that stromal populations remodel, undergoing transcriptional reprogramming in response to tumor-derived cues ***prior*** to metastasis (39), yet the cues responsible for these adaptations remained unclear. Here, we show that tumor-derived LA can induce FRC transcriptional reprogramming of mitochondrial and activation pathways, which are key to FRC function.

Acidification and lactate secretion in the tumor microenvironment is associated with cancer progression, metastasis (34, 53), and therapeutic resistance (54). However, a direct link between LA, pH, and the establishment of a premetastatic niche in lymph nodes has not yet been identified. Our data indicate that LA secreted by tumor cells impacts not only the local tumor, but also the TDLN. We show that lactate and proton uptake by FRCs led to acquisition of an activation signature and altered mitochondrial structure coupled to reduced function, likely mediated by a change in intracellular metabolites and pH. Similarly, CAFs at the tumor exhibiting altered activation and metabolic profiles are associated with metastasis (55–57). Within the tumor microenvironment, LA has been shown to induce a protumor M2-like phenotype in stromal macrophages, inducing Hif1α-driven VEGF expression and angiogenesis (13, 28). This process was dependent on uptake and conversion of lactate to pyruvate by LDH, leading to prolylhydroxylase inhibition and Hif1α protein stabilization (28). In contrast, LA–induced alterations of TDLN FRCs were not Hif1α-dependent. These differences derive from several factors; Colegio and colleagues used concentrations of up to 40-mmol/L LA compared with 15 to 20 mmol/L measured and used in the present study (13), and Sonveaux and colleagues used lactate sodium salts to buffer media at pH 7.3, hence any pH effect of LA would not be apparent (28). Instead, Hif1α dependency is likely to be context- and cell-type–specific.

Consistent with our previous study describing altered immune populations and survival in TDLNs, Brand and colleagues found that LA accumulation in the melanoma tumor microenvironment leads to impaired function and survival of T and NK cells via intracellular acidification, contributing to tumor immune escape and worse overall survival (33). Similarly, a graft-versus-leukemia study revealed that leukemia-derived LA reduced the intracellular pH of T cells, impairing glycolysis and proliferation. Restoration of intracellular pH and metabolite concentrations with sodium bicarbonate treatment reversed these effects, and enhanced graft-versus-leukemia T-cell activity (58).

Other work links mitochondrial dynamics with T-cell fate (59); memory T cells possessed fused mitochondria (elongated, tubular mitochondria that function) whereas effector T cells (T_E_) had fissed mitochondria (punctate mitochondria that lead to imbalanced redox). Consistent with our FRC data, the punctate mitochondrial phenotype of T_E_ cells coincided with increased incorporation of MTG, decreased OCR and spare respiratory capacity (SRC). We also note that effects we observed in TDLNs may also be amplified by locally produced LA, with research indicating that during immune responses, LA released from proliferating lymph node leukocytes creates localized acidic pockets to regulate T-cell activity (60).

Previously, we showed that FRCs of TDLNs become more activated, reminiscent of CAFs and fibroblasts in fibrotic disease. This was evident by increases in Pdpn and Thy1 expression, but also by increased collagen production and contractility. Measured in high quantities in TCM and within tumors *in vivo*, we identified LA as a driver of Pdpn and Thy1 expression, and collagen production by FRCs in draining lymph nodes in this study. Although direct sampling and quantification of lactate within TDLNs *in vivo* was variable, the trends observed warrant further investigation. Indeed, a peripheral subcutaneous bolus of TCM or LA alone was sufficient to induce the same FRC activation signatures in draining nodes, as measured in TDLNs (39), *in vitro* and with TCM doses *in vivo*. Consistent with this, lactate has been shown to induce collagen genesis in fibroblasts (61) and myofibroblast differentiation in idiopathic pulmonary fibrosis (IPF; ref. 62). In IPF lungs, LA accumulation and LDH5 expression activated TGFβ via a pH-dependent mechanism to induce myofibroblast differentiation, coincident with significant ECM deposition.

In addition to altered FRC activation states, we observed mitochondrial adaptations in TCM- and LA–treated FRCs. Of note, although LA induced significant changes in these two functional pathways, not all tumor-induced genes/metabolite alterations in FRCs were driven by LA alone. This likely reflects the complex composition of tumor-derived factors reaching lymph nodes and the diversity of factors contained within. Reduced oxidative phosphorylation and decreased glycolytic activity with increased uptake of LA and pyruvate from culture medium were also measured. However, instead of fueling the TCA cycle we identified an increase towards acylcarnitine and citrate, further processing to α-ketoglutarate and glutamate, but little processing towards succinate, fumarate, or malate. Acylcarnitines are essential for the transport of fatty acids into mitochondria for β-oxidation and for CoA homeostasis. They thus provide indirect evidence of altered mitochondrial metabolism (63) and indicate a dysfunction of the TCA cycle. Acylcarnitines and citrate have further been shown to modulate histone acetylation in the nucleus and to impact epigenetic reprogramming by balancing acetyl-CoA levels (64, 65). Hence, increases in acylcarnitine and citrate levels in FRCs could contribute to the observed changes in gene expression. Although we observed a reduction in FRC OCR with LA treatment, the mechanisms driving this change have yet to be determined and warrant further investigation. Deeper examination of lipid metabolism and the effects of an altered TCA on electron transport chain activity in FRCs may provide greater insight into the pathways responsible.

Our study highlights that tumor-derived metabolites might alter lymph that bathes downstream TDLNs, yet we still know relatively little about the impact of a changed lymph composition on lymph-node function. Alterations in lymph-borne components such as bile salts, glucose, pyruvate, and trigycerols could have widespread functional consequences. Indeed, increased levels of bile acid in lymph node metastases—either from the lymph node microenvironment or tumor cells—supports metastatic colonization of lymph nodes via yes-associated protein (66). Additionally, cytokines such as IL17 support a switch in FRC status from quiescent to metabolically fit (67).

To summarize, the present study set out to define tumor-derived factors responsible for stromal adaptations within premetastatic TDLNs. It is clear that no single factor is responsible for all tumor-induced effects on FRCs. While excluding mechanical cues such as elevated fluid drainage, or proteins and nucleic acid, we identified lactic acid as one of the major factors inducing FRC reprogramming towards a more activated and metabolically altered status. The functional changes induced in these cells highlights the potential benefit of targeting tumor acidity and/or LA accumulation at both the tumor and connected TDLN.

## Supplementary Material

Supplementary Figure
