# Supplementary material for: Tumor-Derived Lactic Acid Modulates Activation and Metabolic Status of Draining Lymph Node Stroma
Source: Cancer Immunol Res. 2022 Mar 9;10(4):482–97. doi: 10.1158/2326-6066.CIR-21-0778 (PMC7612582; doi:10.1158/2326-6066.CIR-21-0778)
Supplement: Supplementary Figure [file cir-21-0778_supplementary_figures_1-3_supps1-3.pdf]

Supp Fig 1

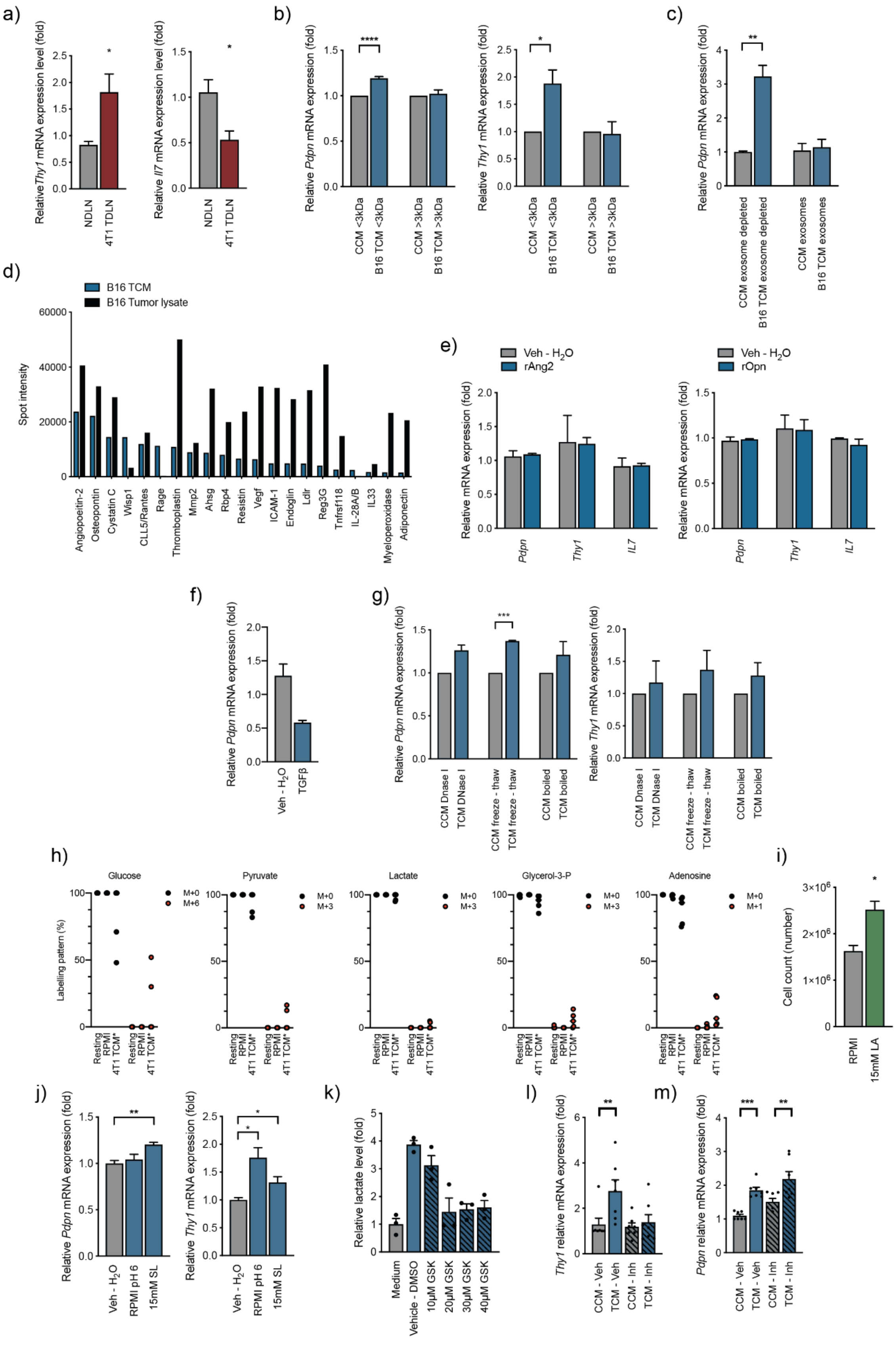

a)
Relative Thy1 mRNA expression level (fold)
Relative Il7 mRNA expression level (fold)
NDLN
4T1 TDLN
b)
Relative Pdpn mRNA expression (fold)
Relative Thy1 mRNA expression (fold)
CCM <3kDa
B16 TCM <3kDa
CCM >3kDa
B16 TCM >3kDa
c)
Relative Pdpn mRNA expression (fold)
CCM exosome depleted
B16 TCM exosome depleted
CCM exosomes
B16 TCM exosomes
d)
B16 TCM
B16 Tumor lysate
Spot intensity
Angiopoeitin-2
Osteopontin
Cystatin C
Wisp1
CLL5/Rantes
Rage
Thromboplastin
Mmp2
Ahsg
Rbp4
Resistin
Vegf
ICAM-1
Endoglin
Ldlr
Reg3G
Tnfrsf118
IL-28A/B
IL33
Myeloperoxidase
Adiponectin
e)
Veh - H2O
rAng2
rOpn
Relative mRNA expression (fold)
Pdpn
Thy1
IL7
f)
Relative Pdpn mRNA expression (fold)
Veh - H2O
TGFβ
g)
Relative Pdpn mRNA expression (fold)
Relative Thy1 mRNA expression (fold)
CCM Dnase I
TCM DNase I
CCM freeze - thaw
TCM freeze - thaw
CCM boiled
TCM boiled
h)
Glucose
Pyruvate
Lactate
Glycerol-3-P
Adenosine
Labelling pattern (%)
M+0
M+6
M+3
M+1
Resting RPMI
4T1 TCM*
i)
Cell count (number)
RPMI
15mM LA
j)
Relative Pdpn mRNA expression (fold)
Relative Thy1 mRNA expression (fold)
Veh - H2O
RPMI pH 6
15mM SL
k)
Relative lactate level (fold)
Medium
Vehicle - DMSO
10μM GSK
20μM GSK
30μM GSK
40μM GSK
l)
Thy1 relative mRNA expression (fold)
CCM - Veh
TCM - Veh
CCM - Inh
TCM - Inh
m)
Pdpn relative mRNA expression (fold)

Supplementary figure S1. Factor depletion, sodium lactate and low pH medium controls, and proliferation assay.

(**A**) Quantitative RT-PCR analysis of *Thy1* (left) and *Il7* (right) in non-draining lymph nodes (NDLNs) and tumor-draining LNs (TDLNs) from 4T1 orthotopically injected, female Balb/c mice. n = 2 independent biological experiments with 2-3 replicates.

(**B**) Quantitative RT-PCR analysis of *Pdpn* (left) and *Thy1* (right) in FRCs cultured *in vitro* and treated for 4 days with control conditioned medium (CCM) or B16.F10 tumor conditioned medium (B16 TCM) with either medium at <3kDa or >3kDa. n = 4 independent biological experiments.

(**C**) Quantitative RT-PCR analysis of *Pdpn* in *in vitro* cultured FRCs treated for 4 days with exosome depleted CCM or TCM or with exosomes isolated from CCM or B16 TCM. n = 2-5 independent biological experiments.

(**D**) Cytokine array of B16 TCM and B16 tumor lysates showing the top 21 detected cytokines as measured by spot intensity. n = 1

(**E**) Quantitative RT-PCR analysis of *Pdpn*, *Thy1*, and *Il7* in *in vitro* cultured FRCs treated for 4 days with 1 µg/ml recombinant murine angiopoietin 2 (rAng2; left) or 5 µg/ml recombinant murine osteopontin (rOPN; right). n = 2 independent biological experiments with 3 replicates.

(**F**) Quantitative RT-PCR analysis of *Pdpn* in *in vitro* cultured FRCs treated for 4 days with 2 ng/ml TGFβ. n = 3 independent biological experiments.

(**G**) Quantitative RT-PCR analysis of *Pdpn* (left) and *Thy1* (right) in *in vitro* cultured FRCs treated for 4 days with CCM or TCM subjected to DNAse I treatment, freeze-thaw cycles or boiling. n = 2 independent biological experiments.

(**H**) Labeling pattern of metabolites of whole tissues of brachial LNs. TCM of ^{13}C-glucose labeled 4T1 tumor cells (4T1 TCM*) was injected subcutaneously every day for 11 days at the shoulder in lieu of a tumor *in vivo*. Metabolites were measured using LS-MS. 'M+n' is the molecular mass plus the number of incorporated heavy carbons. n = 1 experiment with 5 LNs.

(**I**) Total LN cells in draining LNs from female C57Bl/6 mice assessed by flow cytometry injected over 11 days subcutaneously each day with 15mM LA in RPMI. Mean with SD. n = 1 experiment with 3 LNs.

(**J**) Quantitative RT-PCR analysis of *Pdpn* (left) and *Thy1* (right) in FRCs cultured *in vitro* and treated with Vehicle (Veh – H_2O), RPMI at pH 6 or 15mM sodium lactate (SL) for 4 days. n = 3 independent biological experiments.

(**K**) Relative lactate levels in TCM of B16.F10 treated for 24h with a Ldha/ Ldhb inhibitor (GSK 2837808A). n = 3 independent biological experiments.

(**L**) Quantitative RT-PCR analysis of *Thy1* in FRCs cultured *in vitro* and cultivated with TCM of B16.F10 tumor cells treated with either vehicle (DMSO, Veh) or 40µM Inhibitor (Inh) for 4 days. n = 4 independent biological experiments with 1-2 replicates.

(**M**) Same as in (**L**), but Quantitative RT-PCR analysis of *Pdpn.*

All data are means with SEM from biological independent replicates (unless stated differently). Significance (**P*<0.05, ***P*<0.01, ****P*<0.001 and *****P*<0.0001) was determined by unpaired two-tailed *t*-test.

Supp Fig 2

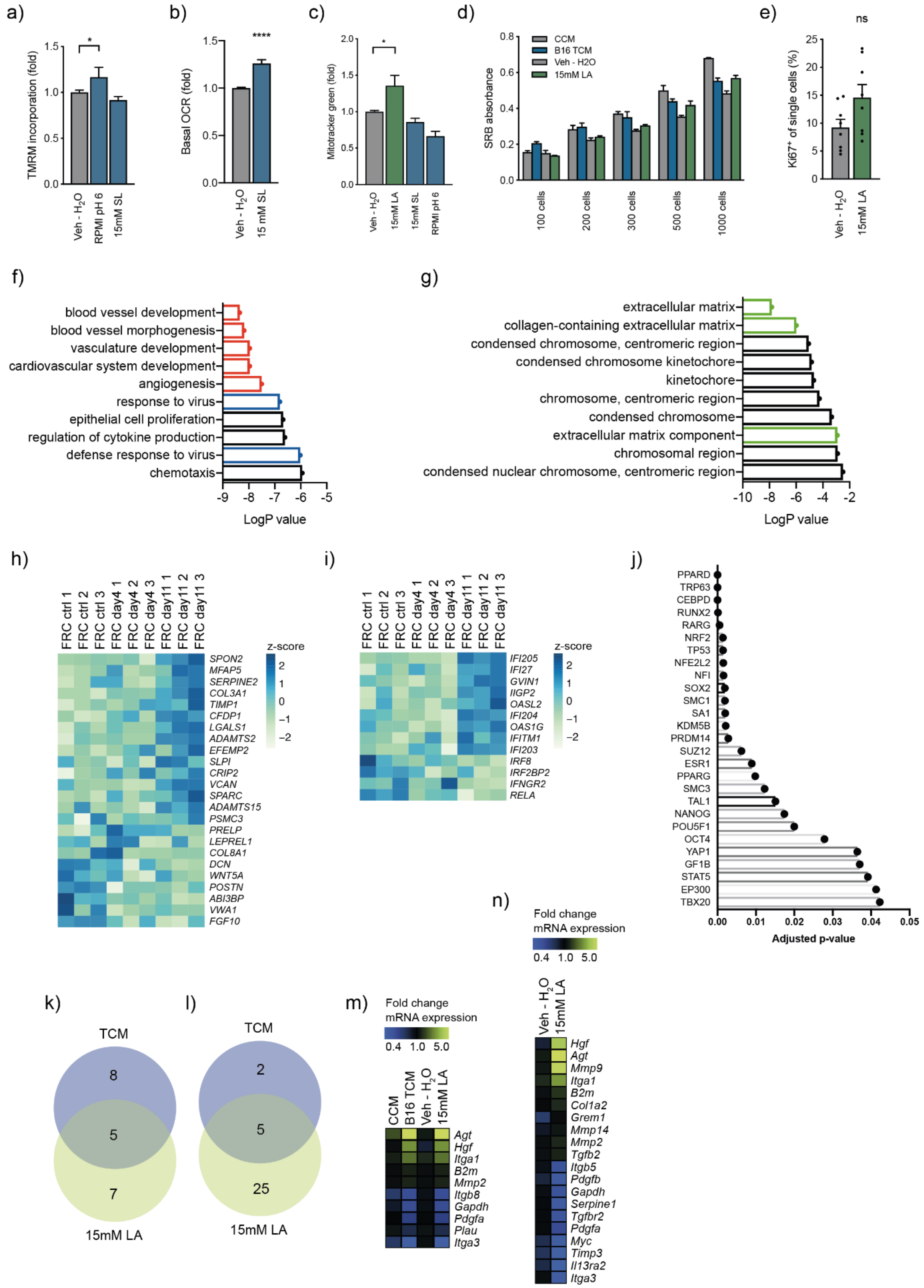

a)
TMRM incorporation (fold)
Veh - H2O
RPMI pH 6
15mM SL
b)
Basal OCR (fold)
Veh - H2O
15 mM SL
c)
Mitotracker green (fold)
Veh - H2O
15mM LA
15mM SL
RPMI pH 6
d)
SRB absorbance
CCM
B16 TCM
Veh - H2O
15mM LA
100 cells
200 cells
300 cells
500 cells
1000 cells
e)
ns
Ki67+ of single cells (%)
Veh - H2O
15mM LA
f)
blood vessel development
blood vessel morphogenesis
vasculature development
cardiovascular system development
angiogenesis
response to virus
epithelial cell proliferation
regulation of cytokine production
defense response to virus
chemotaxis
LogP value
g)
extracellular matrix
collagen-containing extracellular matrix
condensed chromosome, centromeric region
condensed chromosome kinetochore
kinetochore
chromosome, centromeric region
condensed chromosome
extracellular matrix component
chromosomal region
condensed nuclear chromosome, centromeric region
LogP value
h)
FRC ctrl 1
FRC ctrl 2
FRC ctrl 3
FRC day4 1
FRC day4 2
FRC day4 3
FRC day11 1
FRC day11 2
FRC day11 3
z-score
SPON2
MFAP5
SERPINE2
COL3A1
TIMP1
CFDP1
LGALS1
ADAMTS2
EFEMP2
SLPI
CRIP2
VCAN
SPARC
ADAMTS15
PSMC3
PRELP
LEPREL1
COL8A1
DCN
WNT5A
POSTN
ABI3BP
VWA1
FGF10
i)
IFI205
IFI27
GVIN1
IIGP2
OASL2
IFI204
OAS1G
IFITM1
IFI203
IRF8
IRF2BP2
IFNGR2
RELA
j)
PPARD
TRP63
CEBPD
RUNX2
RARG
NRF2
TP53
NFE2L2
NFI
SOX2
SMC1
SA1
KDM5B
PRDM14
SUZ12
ESR1
PPARG
SMC3
TAL1
NANOG
POU5F1
OCT4
YAP1
GF1B
STAT5
EP300
TBX20
Adjusted p-value
k)
TCM
8
5
7
15mM LA
l)
TCM
2
5
25
15mM LA
m)
Fold change mRNA expression
0.4 1.0 5.0
CCM
B16 TCM
Veh - H2O
15mM LA
Agt
Hgf
Itga1
B2m
Mmp2
Itgb8
Gapdh
Pdgfa
Plau
Itga3
n)
Fold change mRNA expression
0.4 1.0 5.0
Veh - H2O
15mM LA
Hgf
Agt
Mmp9
Itga1
B2m
Col1a2
Grem1
Mmp14
Mmp2
Tgfb2
Itgb5
Pdgfb
Gapdh
Serpine1
Tgfbr2
Pdgfa
Myc
Timp3
Il13ra2
Itga3

Supplementary figure S2. Transcriptional analyses of FRCs.

(**A**) *In vitro* FRCs treated for 4 days as in (**I**), stained with TMRM and analyzed by flow cytometry. n = 3 independent biological experiments with 1-3 replicates.

(**B**) Baseline OCR from vehicle (Veh – H_2O) and 15mM sodium lactate (SL) treated FRCs. n = 3-4 independent biological experiments with each 5-replicates.

(**C**) *In vitro* FRCs treated for 4 days with vehicle (Veh – H_2O), 15mM LA, 15mM sodium lactate (SL), or RPMI at pH 6, stained with Mitotracker green and analyzed by flow cytometry. n = 3 independent biological experiments with 1-2 replicates.

(**D**) SRB proliferation assay of *in vitro* cultured FRCs treated for 4 days with CCM, B16 TCM, vehicle (Veh – H_2O) or 15mM LA and seeded in a 96-well plate at different starting cell numbers. n = 1 with 3 replicates. Mean with SD.

(**E**) *In vitro* FRCs treated for 4 days with vehicle (Veh – H_2O) or 15mM LA, stained for Ki67 and analyzed by flow cytometry. n = 3 independent biological experiments with 2-3 replicates.

(**F**) Top 10 most significant gene ontology (GO) terms for 'biological process'. All blood vessel development processes marked in red, all response to virus in blue.

(**G**) Top 10 most significant gene ontology (GO) terms for 'cellular component'. All extracellular matrix processes marked in green.

(**H**) Extracellular matrix genes deregulated in *ex vivo* sorted FRCs of tumor-draining lymph nodes (TDLNs; Riedel et al., 2016)

(**I**) Interferon related genes deregulated in *ex vivo* sorted FRCs of TDLNs (Riedel et al., 2016)

(**J**) Transcription factor (TF) analysis displaying TFs with adjusted $P<0.05$ (Enrichr, ChEA 2016) using all up and down regulated genes in 15mM LA to Veh – H_2O.

(**K**) Overlap of genes significantly ($P<0.05$) upregulated covered by the PCR array (PAMM-120ZG, Mouse Fibrosis, QIAGEN) in comparisons between B16 TCM to CCM or 15mM LA to vehicle (Veh – H_2O). FRCs were treated for 4 days and each sample was run 5 times from 5 independent biological replicates.

(**L**) Overlap of genes significantly ($P<0.05$) downregulated in comparisons as in (**A**).

(**M**) Heatmap depicting the 10 commonly deregulated genes between B16 TCM to CCM and 15mM LA to vehicle (Veh – H_2O) as average of fold changes of mRNA expression.

(**N**) Heatmap depicting the top 10 up- and downregulated genes between 15mM LA to vehicle (Veh – H_2O) as average of fold changes of mRNA expression.

All data are means with SEM from biological independent replicates (unless stated differently). Significance (*$P<0.05$, **$P<0.01$, ***$P<0.001$ and ****$P<0.0001$) was determined by unpaired two-tailed *t*-test.

Supp Fig 3

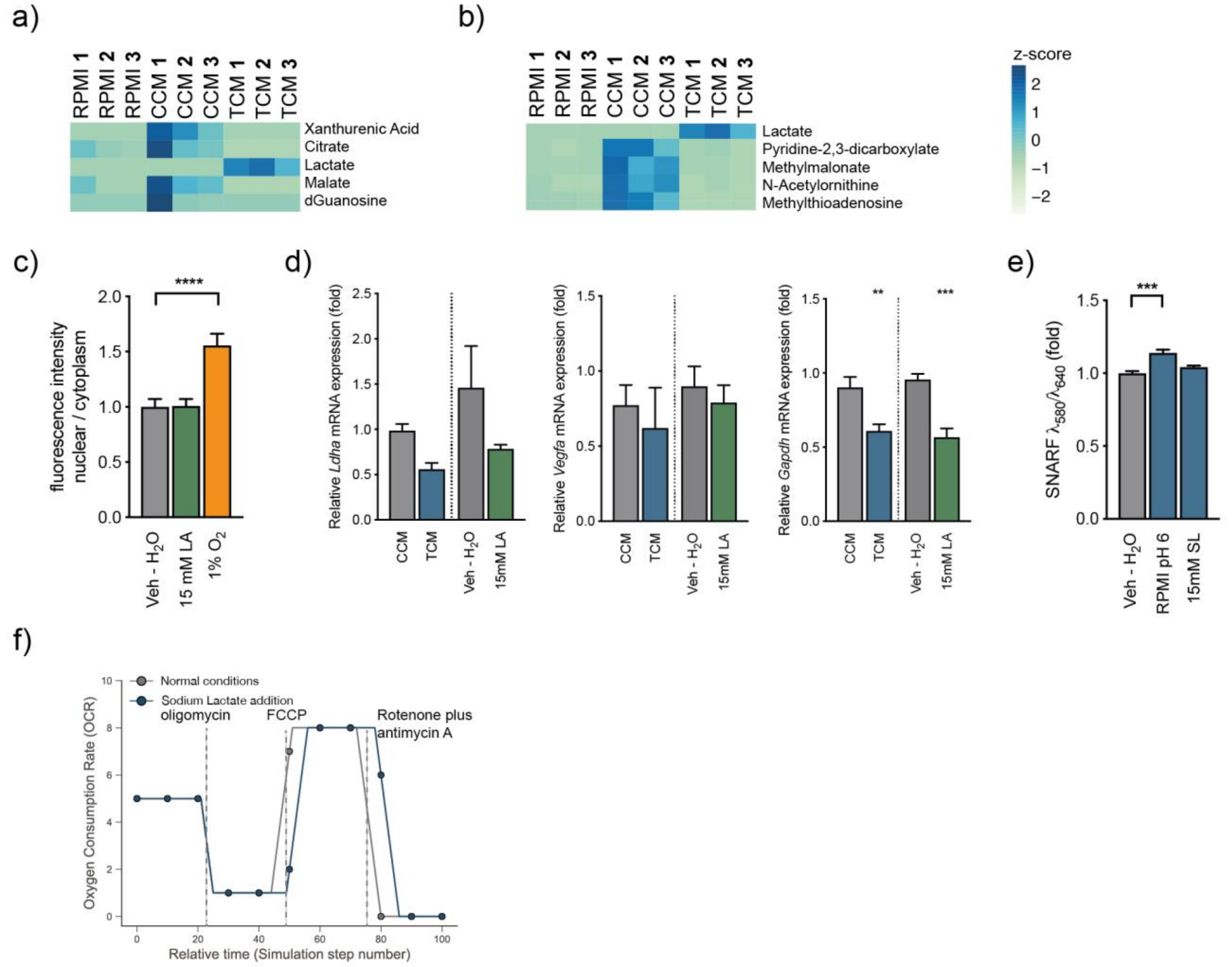

Supplementary figure S3. pH regulates metabolic shift.

(**A**) Metabolites in same order as in (**Fig 4c**), but measured in the respective conditioned medium (CCM or TCM) measured by LC-MS.

(**B**) Significant (*P*<0.05) metabolites in TCM vs CCM measured by LC-MS.

(**C**) Quantification of confocal fluorescent images stained for Hif1a and nuclei. Fluorescent intensity was calculated as nuclear / cytoplasm and was normalized on cell number. n = 3 independent biological experiments with each 8-10 fields of view.

(**D**) Quantitative RT-PCR analysis of *Ldha* (left), *Gapdh* (middle) and *Vegfa* (right) in *in vitro* cultured FRCs treated for 4 days with CCM, B16 TCM, vehicle (Veh – H_2O) or 15mM LA. n = 2-5 independent biological experiments.

(**E**) Intracellular pH of FRCs treated for 4 days with vehicle (Veh – H_2O), RPMI at a pH of 6 (RPMI pH 6), or 15mM sodium lactate (SL) and stained with SNARF. n = 2-6 independent biological experiments.

(**F**) Metabolic analysis of mitochondrial function in the computational model. Shown is the OCR for mitochondria exposed to sodium lactate compared to control cells.

All data are means with SEM from biological independent replicates (unless stated differently). Significance (**P*<0.05, ***P*<0.01, ****P*<0.001 and *****P*<0.0001) was determined by unpaired two-tailed *t*-test.
